# Dual-platform metagenomic surveillance distinguishes pathogen and resistome hotspots across agricultural and mixed-use watersheds

**DOI:** 10.1016/j.onehlt.2026.101384

**Published:** 2026-03-08

**Authors:** Yichao Shi, Haley Sanderson, Jiacheng Chuan, Izhar U.H. Khan, Mark Sunohara, Emilia Craiovan, David R. Lapen, Moussa Diarra, Wen Chen

**Affiliations:** a Ottawa Research & Development Centre, Science & Technology Branch, Agriculture & Agri-Food Canada (AAFC), 960 Carling Avenue, Ottawa, ON K1A 0C6, Canada; b Biological Informatics Center of Excellence, Information Systems Branch, AAFC, 107 Science Pl, Saskatoon, SK S7N 5E2, Canada; c The Charlottetown Laboratory, Canadian Food & Inspection Agency (CFIA), 93 Mt Edward Rd, Charlottetown, PEI C1A 5T1, Canada; d Guelph Research and Development Centre, AAFC, 93 Stone Rd W, Guelph, ON N1G 5C9, Canada; e Department of Biology, University of Ottawa, Marie-Curie Private, Ottawa, ON K1N 9A7, Canada

**Keywords:** Freshwater microbiome, agricultural watersheds, metagenomic sequencings, pathogens, antimicrobial resistance

## Abstract

Freshwater systems embedded in agricultural landscapes serve as dynamic reservoirs and conduits for fecal-associated microbes, zoonotic pathogens, and antimicrobial resistance (ARG) and virulence factor (VF) genes. Yet factors that govern their densities and diversity remain a research challenge. From 2016 to 2021, we conducted a longitudinal water surveillance in an agriculturally dominated river basin in eastern Ontario, Canada; characterizing fecal-associated bacterial communities using 16S rRNA gene amplicon and shotgun metagenomic sequencing. Agricultural drainage ditches consistently harbored higher fecal-associated bacterial diversity with pronounced seasonal shifts; i.e., higher levels during larger flow periods in spring and fall. Elevated discharge was associated with enrichment of genera containing zoonotic or opportunistic pathogens, such as those in *Pseudomonas*, *Sphingomonas*, and *Massilia*. Conditionally rare taxa (CRTs), although typically low in abundance, accounted for ∼12.6% of all pathogen-associated genera and disproportionately contributed to community turnover, highlighting their role as transient reservoirs of microbial risk. Shotgun metagenomics detected 27 ARGs, primarily at mixed-use sites, and 14 VFs, mainly in agricultural ditches. Clinically relevant β-lactamase genes (e.g., *oxa*, *imp*, *sme*) co-occurred with metal-resistance operons, a pattern suggestive of possible co-selection, although selective agents were not directly measured. Although the prevalence of ARG and VF was low (<5% of samples), their ecological context indicates potential transmission pathways. Limited overlap in ARGs between short-read and metagenome-assembled genome (MAG)–based profiling reflects their complementary strength: gene-level sensitivity versus host-resolved analysis. Together, these findings demonstrate the utility of integrated amplicon and shotgun metagenomic surveillance for proactive One Health risk assessment in agricultural watersheds.

## Highlights

• Amplicon profiling resolves ecological gradients driven by land use.
• Shotgun sequencing detects clinical pathogens missed by amplicon methods.
• Conditionally rare taxa act as sentinels for pathogen persistence.
• Agri-ditches and mixed-use sites show distinct virulence and resistome hotspots.
• Dual-platform monitoring enables robust One Health risk surveillance.

## Graphical Abstract

**Figure:**
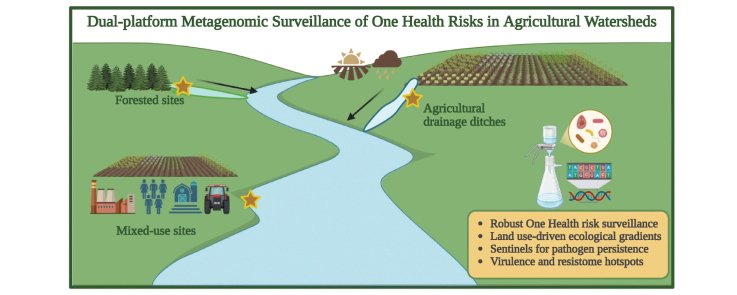

## 1 Introduction

The One Health framework emphasizes the interconnectedness of human, animal, and environmental health, aiming to improve, for example, outcomes and challenges as associated with microbial pathogen emergence and antimicrobial resistance (AMR) through coordinated, cross-sectoral action [1], [2]. Zoonotic pathogens, which account for approximately 60% of infectious diseases in humans, can harbor and disseminate AMR genes and virulence factors (VFs), posing significant public health risks [3]. The widespread use of antibiotics in animal husbandry exerts strong selective pressure on gut microbiota, facilitating the emergence and spread of antibiotic-resistant bacteria (ARBs) [4]. These ARBs can further transfer their resistance genes among native flora through horizontal gene transfer events, vectors, and quorum sensing mechanisms [5]. Moreover, unmetabolized antibiotics often enter the environment and accumulate in soils, sediments, and aquatic systems, where they promote the persistence and propagation of resistance [6]. Consequently, aquatic environments receiving inputs from agricultural runoff and drainage, livestock operations, and municipal wastewater [7], [8], can concurrently act as reservoirs and sources for AMR, VFs, and fecal-associated pathogens, amplifying exposure risks for humans and animals [9], [10], [11].

Aquatic systems are highly dynamic, and their microbial composition and resistome profiles are strongly shaped by land use. Agricultural landscapes with intensive livestock production and/or seasonal manure applications to land, are especially susceptible to AMR gene (ARGs) accumulation and the proliferation of fecal-associated zoonotic pathogens, including *Escherichia coli* O157:H7, *Salmonella* spp., and *Campylobacter* spp. [12], [13], [14]. Urban and other anthropogenic land uses further compound selective pressures through wastewater and industrial effluent discharges [15], [16], [17] for example. Importantly, resistance and virulence determinants are also naturally present in relatively undisturbed ecosystems, where they provide ecological functions within competition, stress tolerance, and survival niches [18]. Monitoring ARGs, VFs, and fecal-associated pathogens in variably influenced waterways helps identify hotspots of contamination and to establish ecological baselines that distinguish anthropogenic impacts from natural background levels [19].

Conventional monitoring approaches, such as culture-based assays and quantitative PCR (qPCR), are valuable for detecting specific fecal indicator bacteria (FIB) and targeted pathogens, ARGs or VF genes. However, such methods capture only a fraction of microbial diversity which can overlook broader ecological interactions and environmental drivers [20], [21], [22]. In Canadian watersheds, studies have shown that pathogen and ARG distributions are shaped by land use and hydrology [23], [24], yet most investigations are constrained by limited spatial or temporal coverage, impeding comprehensive landscape-scale understanding. Advancements in high-throughput sequencing technologies, including both the amplicon-based and shotgun metagenomics, have transformed microbial ecology by enabling comprehensive, systematic snapshots of microbial diversity, taxonomic composition, and functional potential over time in complex environments [25], [26]. Previous studies have shown that combining both approaches was essential to fully capture microbial and functional diversity, in thermal spring ecosystems for example, because each provides complementary insights [27]. The 16S rRNA gene metabarcoding enables sensitive and cost-effective taxonomic profiling of bacteria, making it well-suited for large-scale ecological observatories and detecting compositional shifts across environmental gradients [28], [29]. In contrast, shotgun metagenomics provides an untargeted, genome-wide view of community DNA, allowing simultaneous characterization of ARGs, VFs, mobile genetic elements, and broader functional attributes [30].

While both amplicon and shotgun metagenomic approaches have proven invaluable in environmental microbiome research, each entails methodological limitations that shape data interpretation. Amplicon-based approach, though sensitive and scalable, can be affected by primer bias, limited taxonomic resolution, and constrained functional inference [31]. Shotgun metagenomics, while functionally comprehensive, can yield community structure patterns that differ from marker-gene profiling due to uneven genome coverage, reference database limitations, and taxonomic classification challenges, particularly at finer taxonomic resolutions [30], [32], [33]. Linking functional genes to their taxonomic origins also remains difficult due to assembly fragmentation, horizontal gene transfer (HGT), and incomplete reference databases [25], [34], [35]. Both approaches share a common limitation in resolving bacterial communities at the species level. In short-read 16S rRNA gene sequencing, the conserved nature of the amplified region constrains taxonomic resolution, often preventing reliable differentiation among closely related species within the same genus [36]. Similarly, in shallow shotgun metagenomics, species-level classification is hindered by incomplete reference genomes, HGT, and sequence conservation across taxa [34], [37]. These challenges underscore the importance of cautious interpretation and, when feasible, the integration of complementary targeted methods such as qPCR to improve pathogen identification accuracy.

Despite substantial advances in microbial ecology, significant knowledge gaps remain. Long-term, multi-seasonal investigations that link microbial dynamics to land use pressures, environmental gradients, as well as targeted assessments of the interplay between core and conditionally rare taxa as ecosystem health indicators are still limited [38], [39], [40]. Moreover, few studies have concurrently examined fecal-associated pathogens and ARGs across landscapes using integrated multi-omic frameworks, though global, regional, and watershed-scale investigations have revealed strong associations between anthropological interventions, such as agricultural and urban developments, and fecal pollution, pathogen dissemination, and ARG dynamics [41], [42], [43]. To address these gaps, we conducted a five-year longitudinal study (2016–2021) within an agriculturally dominated but mixed-use river basin in eastern Ontario, Canada. Combining 16S rRNA gene amplicon sequencing and shotgun metagenomic sequencing, we characterized fecal-associated bacterial communities, resistomes, and virulomes across spatial and temporal gradients in surface waters.

This study was guided by the central question: *How do land use and environmental conditions shape the diversity and structure of fecal-associated bacterial communities, particularly the dynamics of potential zoonotic pathogens, ARGs and VFs, in agricultural and mixed-use freshwater ecosystems?* To address this overarching question, we pursued four specific objectives: 1) characterize how land use and seasonal conditions influence the diversity and structure of fecal-associated microbiota, with emphasis on the ecological roles of core and conditionally rare taxa (CRT) in freshwater pathogen reservoirs as quantified health indicators; 2) compare the resolution of 16S rRNA gene amplicon sequencing and shotgun metagenomics for profiling fecal-associated communities and detecting potential pathogens; 3) assess the spatial and temporal dynamics of bacterial genera containing pathogenic species; and 4) map the distribution of ARGs and VFs across land use types to identify their environmental hotspots. Collectively, this study endeavors to advance our understanding of fecal contamination and environmental resistome and virulome determinants in agroecosystems, and by extension, contribute to the advancement of risk-factor based microbial water quality surveillance within a One Health framework.

## 2 Materials & methods

### 2.1 Study site and sampling design

This study was conducted as part of the Environmental Change OneHealth Observatory (ECO^2^), a long-term field-based research initiative focused on natural capital in agroecosystems and its influence on animal, environment, and public health, with due consideration of maintaining or augmenting agricultural productivity [44], [45]. Fieldwork was conducted in the South Nation River (SNR) basin in eastern Ontario, Canada, which covers approximately 3,900 km^2^. Land use in the basin is primarily agricultural (>60% of land area), including dairy farming operations and associated field cropping systems. Stream sampling locations were selected to represent gradients in upstream land use and stream order [38].

**Fig. 1:**
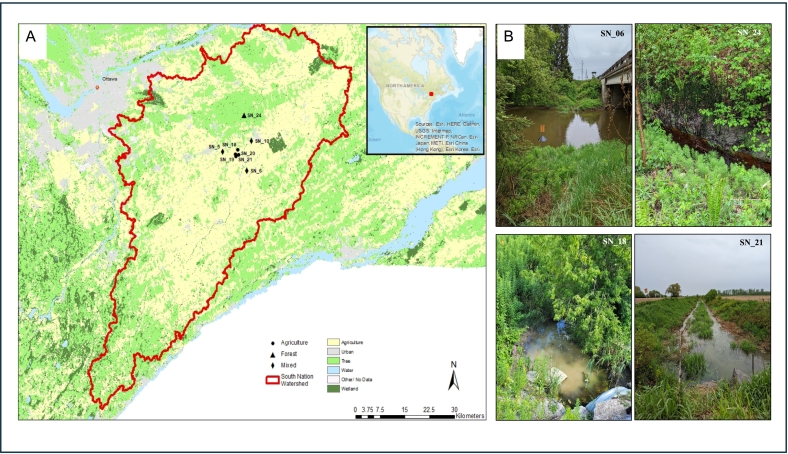
Geological map of the study site (A) and photographs of representative sampling locations across each land use type (B).

This study included eight water sampling sites representing different upstream land uses (Fig. 1). Four agricultural drainage ditch sampling sites (SN_18, SN_19, SN_20, and SN_21), representative of the ubiquity of such features in the region, have been documented in earlier studies [39], [44]. Sites SN_18 and SN_20 were monitored continuously from 2016 to 2021, while SN_19 and SN_21 were included since 2017. The first ditch (SN_18 and SN_19) remained unmanaged, whereas the second (SN_20 and SN_21) underwent vegetation brushing in February 2018 followed by channel dredging in November 2018 [44]. The managed and unmanaged ditches drain catchment areas are 225 ha and 467 ha, respectively [46]. The drainage ditches are shallow, open-channel systems with nominal water depths around 0.2 to 0.5m (occasionally 1m during high rainfall) depth. Across the 2016–2021 monitoring period for all ditch sites, specific conductivity averaged 0.84 mS cm^-1^ (range: 0.38–2.27 mS cm^-1^), and turbidity averaged 46 NTU (range: 0.5–944 NTU). While these site pairs served as upstream-downstream monitoring points within their respective watersheds, they were not replicates in the strict sense. Prior to 2018, neither ditch had experienced major maintenance for over 40 years, aside from occasional removal of in-stream debris dams etc. by farmers. Three additional stream monitoring sites (SN_5, SN_6, and SN_10) were influenced by mixed-land uses, which included agriculture, urban development, and forested areas. A single forested reference site (SN_24), situated at outflow of a treed wetland with a <5 km^2^ catchment area, was also included in the analysis as a baseline/control given there were no anthropogenic land uses upstream of the site [45].

Surface water samples were collected biweekly during ice free conditions from April to November over six years (2016–2021). Sampling in the ditches was interrupted during dry-spell periods which induced limited (discontinuous water pools) or no-flow conditions, to avoid sample contamination by sediments. Due to logistical constraints imposed by the COVID-19 pandemic, only a limited number of samples were collected in 2020; however, a total of 499 samples were collected across the eight sampling sites over the entire study period. Samples were collected from 0–50 cm depth (avoiding sediment in sample), transported on ice, and processed within 24 hours at the Ottawa Research and Development Centre, Agriculture and Agri-Food Canada (AAFC). Water physicochemical parameters and land use characteristics followed established methods [39]. On-site measurements included water temperature, pH, dissolved oxygen, specific conductivity, and turbidity. These parameters were measured using YSI multi-parameters sondes; a YSI 6600 multi-parameter sonde was used from 2016 to 2018 and a YSI ProDSS multi-parameter digital water quality meter was used from 2019 to 2021. Nutrient analyses (e.g., ammonia, nitrite, nitrate, total Kjeldahl nitrogen, dissolved reactive phosphorus, and total phosphorus) were conducted at the City of Ottawa Laboratory Services located at the Robert O. Pickard Environmental Centre (Ottawa, ON). Organic carbon (total and dissolved) was measured via APHA Method 2540D [47].

Land use and stream order were determined using GIS-based catchment analyses (2, 5, and 10 km upstream) following Wilkes et al. [48] and Lyautey et al. [49]. Meteorological data (air temperature, precipitation, solar radiation) were recorded near SN_20 using a HOBO data logger (Bourne, MA, USA) from 2016 to 2018 and then a Forest Technology Services (FTS) weather data logger (Victoria, BC, Canada) from 2018 to 2021, with metrics calculated for the day of sampling and up to seven days prior. Due to differences in stream order among the sites, river discharge data for the Castor River (Russell Station 02LB006) were retrieved from the Water Survey of Canada [50] as a standardized streamflow proxy as per approach described in [50].

### 2.2 DNA extraction, amplicon, and shotgun metagenomic sequencing

To capture a broad range of microbial taxa, including bacteria and fungi, 500 mL of each surface water sample underwent sequential filtration. Samples were first passed through 0.7 μm borosilicate glass fibre filters (Thermo Fisher, Ottawa, ON) to retain larger particles and fungal spores, followed by filtration through sterile 0.22 μm nitrocellulose membranes (Millipore, Billerica, MA, USA) to collect smaller microbial cells such as bacteria and archaea. DNA was extracted from both filter types using the DNeasy PowerSoil® Kit (Qiagen), and quantified with a Qubit 3.0 fluorometer (Invitrogen). DNA integrity was verified by 1% agarose gel electrophoresis, and samples were stored at –80 °C until sequencing.

For amplicon sequencing, DNA from both filter types was used to amplify the V4–V5 region of the 16S rRNA gene with primers 515F-Y and 926R. PCR was conducted using Qiagen HotStar MasterMix under standard cycling conditions (95 °C for 3 min; 25 cycles of 95 °C for 30 s, 55 °C for 30 s, 72 °C for 30 s; final extension at 72 °C for 5 min). Amplicons were purified (NucleoMag beads, Macherey-Nagel), pooled in equimolar concentrations, and sequenced on an Illumina MiSeq using a 500-cycle v2 kit (2 × 250 bp reads). To ensure consistency with the shotgun metagenomic dataset, only amplicon data derived from the 0.22 μm filters were used for downstream analysis.

Of the 499 total water samples collected, a subset of 270 samples was selected for shotgun metagenomic sequencing. Selection was based on multiple criteria to ensure spatial, temporal, and land use representation across the five-year period. Priority was given to samples with high-quality and high-concentration DNA, as verified by Qubit quantification and agarose gel electrophoresis. Additionally, representative samples were chosen to capture seasonal variability, land use gradients (agriculture, mixed-use, forest), and key hydrological events (e.g., spring runoff, post-harvest). Resource limitations in sequencing capacity and cost also necessitated a focused yet informative subset for deep metagenomic profiling, while ensuring broad coverage of microbial functional potential. Shotgun metagenomic sequencing was performed on DNA from the 0.22 μm filters. Libraries were prepared using the Illumina Nextera XT kit. Samples from 2016–2019 were sequenced on an Illumina HiSeq 2500 (2 × 150 bp) at NRC Montreal, while 2020–2021 samples were sequenced on the NovaSeq 6000 at NRC Saskatoon. No mock communities were included for comparison the recovery between the two instruments. All DNA was stored at –80 °C before and after library preparation to maintain quality.

While mock communities were not included to benchmark recovery efficiency, strict adherence to standardized DNA extraction and library preparation protocols was maintained across all samples to ensure methodological consistency. Consequently, the comparison between platforms relies on the detection of robust ecological patterns within complex environmental matrices rather than synthetic standards.

### 2.3 Data processing for 16S rRNA amplicon sequencing date

Adapter and primer sequences were trimmed from raw FASTQ files using Atria (v4.1.1) [51] and Cutadapt v4.1 [52]. Paired-end reads were processed in QIIME2 [53] using the DADA2 plugin (v1.14) [54] for quality filtering, denoising, chimera removal, and amplicon sequence variant (ASV) inference. Forward and reverse reads were truncated at 229 nt and 187 nt, respectively, based on quality profiles. Taxonomic classification was conducted using the q2-feature-classifier plugin in QIIME2 [53], with a Naive Bayes classifier trained on the Greengenes2 reference database [55]. A minimum bootstrap confidence threshold of 70% was applied for taxonomic assignments.

### 2.4 Data processing for shotgun sequencing data

#### 2.4.1 Short read-based taxonomic profiling and detection of antimicrobial resistance and virulence factor genes

Raw reads from shotgun sequencing were first subjected to adapter removal using BBDuk (https://sourceforge.net/projects/bbmap/), followed by quality trimming with Trimmomatic v0.39 [56] using the parameters: LEADING:3, TRAILING:3, SLIDINGWINDOW:4:15, MINLEN:36, and HEADCROP:6. The quality and quantity of trimmed reads were assessed using FastQC v0.12.1 [57], and results were summarized with MultiQC v1.2 [58].

Taxonomic profiling was performed using Kraken2 v2.1.3 [59] with the May 17, 2021 version of the Kraken2 database, followed by Bracken v2.9 [60] to estimate relative abundances at species, genus, family, and order levels. A pre-built Bracken database, based on the NCBI nucleotide (nt) database dated May 2, 2023, was used with a 150 bp k-mer specification. Post-processing was automated with a Snakemake v7.32.4 [61] pipeline, which parsed and merged Bracken reports, retaining sample ID, taxonomy name, and estimated read counts (new_est_reads column) for downstream analyses. This classification workflow was selected for its computational efficiency on large datasets and its demonstrated high accuracy and sensitivity for pathogen detection and abundance estimation in complex metagenomic communities [62]**.**

Short read-based ARG and VF detection were performed by mapping trimmed reads to the MEGARes v3.0 [63] and VFDB v12-06-2023 (complete set) [64] databases using KMA v1.3.23 [65]. Presence or absence of ARGs, as determined by KMA, was used for downstream analyses.

#### 2.4.2 Assembly of metagenome assembled genomes (MAGs) and detection of their antimicrobial resistance genes and virulence factors

The trimmed FASTQ files were subjected to co-assembly using MEGAHIT v1.2.9, with a minimum contig length of 1 kb [66], [67]. Gene prediction on assembled contigs was performed with Prodigal v2.6.3 [68]. Quality-trimmed reads were then mapped back to the assembled contigs using Bowtie2 v2.4.5 [69], and alignments were stored in BAM files with Samtools v1.7 [70].

Sample-specific contig binning was executed based on tetranucleotide frequencies using CONCOCT v1.1.0 [71] and MetaBAT2 v2.12.1 [72]. Consensus bins were generated with DAS Tool v1.1.3 [73]. MAG completeness and contamination were assessed using CheckM2 v1.0.1 [74], retaining bins with >50% completeness and <10% contamination per MIMAG standards [75], resulting in 41 medium- to high-quality MAGs.

Taxonomic classification was conducted with GTDB-Tk v2.3.2 [76] against GTDB Release R214 [77]. Unclassified bins lacking bacterial or archaeal marker genes were further identified using BLAST v2.15.0 [78] against the NCBI nucleotide database (July 2023), accepting matches with >90% identity and e-value <1e-5.

Antimicrobial resistance genes (ARGs) and virulence factors (VFs) within MAGs were detected by BLAST against MEGARes v3.0 [63] and VFDB v12-06-2023 [64], considering hits with >80% identity. MEGARes hits requiring further SNP analysis to confirm resistance were excluded to allow comparison with read-based ARG detection.

### 2.5 Curated reference databases for fecal-associated and opportunistic pathogen taxa

To characterize fecal-associated bacterial communities, we curated the *Fecal-Associated Microbiome Reference (FAMR)* by integrating genus-level taxonomic information from two established sources: the Animal Gut Microbiome Database (AMDB) [79] and the Annotated Database of Domestic Animal Gut Microbiota (ADDAGMA) [80]. AMDB provides a broad collection of bacterial taxa and samples across diverse animal species, while ADDAGMA focuses specifically on gut microbiomes of four key domestic animals (cattle, horse, pig, and chicken). This combined database enabled the identification of fecal-associated bacterial genera from both 16S rRNA and shotgun metagenomic sequencing datasets.

For opportunistic pathogen detection, we developed an *Opportunistic and Zoonotic Pathogen Reference (OZPR)* curated a separate database by consolidating genus-level taxonomic information from several authoritative sources. The core reference was a list of 1,513 bacterial species with confirmed pathogenicity to humans [81], which was expanded by incorporating species from the NCBI Pathogen Detection database (97 species) [82], the PATRIC pathogen database (https://www.bv-brc.org/) [83], and the Voluntary 2025 U.S. National Animal Health Reporting System (NAHRS) Reportable Diseases, Infections, and Infestations List [84]. We also included taxa from the WHO 2024 bacterial priority pathogen list [85] and the CDC zoonotic pathogen list (https://www.cdc.gov/healthy-pets/diseases/index.html) [86]. The final pathogen reference list comprised 336 genera and was used to extract and compare pathogen-associated taxa from the sequencing data. Because taxonomic resolution from sequencing data is typically limited to the genus level, the OZPR reference includes genera that encompass both pathogenic and commensal species; therefore, detected genera should be interpreted as potentially pathogen-associated rather than definitively pathogenic.

### 2.6 Statistical analysis

All statistical analyses were performed in R (ver. 4.5.1) [87]. Alpha diversity indices were used to quantify within-sample ASV diversity of fecal-associated bacterial communities. The Shannon Index (H), Simpson Index (D), and Chao1 Index were calculated using the vegan (v2.6-6.1) [88] and *biodiversityR* (v2.16-1) [89] packages. Shannon-based True Diversity (Shannon-TD = exp(H)) and Simpson-based True Diversity (Simpson-TD = 1/(1–D)) were computed following the approach described by Jost [90]. Normality of alpha diversity distributions was assessed using the shapiro.test function, and Box-Cox transformations were applied when needed using the *boxcox* function in the MASS package (v7.3-65) [91]. General linear mixed-effects models (GLMMs) were implemented via the *glmmTMB* function in glmmTMB package (v1.1.12) [92], to evaluate the effects of site type, year, and their interactions on alpha diversity and physicochemical properties. Site type and year were treated as fixed effects, block as a random effect, and repeated measures were nested within year-week. Post hoc comparisons were performed using the emmeans package (v1.10.2) [93] with Bonferroni-adjusted P-values. Environmental drivers of diversity were identified via random forest analysis using the randomForest package (v4.7-1.1) [94]. Random Forest regression was selected for its ability to model complex, nonlinear relationships and interactions among environmental variables without assuming normality or linearity, which are common limitations in ecological datasets [95]. Finally, Spearman’s rank-order correlation between Shannon-TD and physicochemical parameters were evaluated using *rcorr.adjust* function in RcmdrMisc (v2.9-1) [96].

To characterize the temporal stability of bacterial communities, we identified core taxa and conditionally rare taxa (CRT) across all sampling sites using methods described by Shade and Stopnisek [97] and Shade et al. [98], with detailed procedures described by Shi et al. [40]. In brief, core ASVs were selected based on abundance–occupancy distributions and their contributions to overall community heterogeneity. CRTs were identified by assessing ASVs with ≥0.1% abundance that exhibited bimodal abundance distributions over time, using a coefficient of bimodality (b) threshold of 0.90 [98]. The percent contributions of core taxa and CRT to overall community beta-diversity were calculated separately for agricultural drainage ditch sites and mixed-use sites, but not for the single forested site (SN_24). To evaluate the effect of land use on the contributions of core taxa and CRT, linear mixed-effects models were used with land use class as a fixed factor and sampling date as a repeated measure. Additionally, to identify key environmental drivers of temporal variation in Shannon-based true diversity, core and CRT contributions to community dissimilarity, variable importance was ranked using the *randomForest* function (randomForest package v4.7–1.1) [94].

The glmmTMB models were used to assess the effect of site_type on robust centered log ratio (rCLR)-transformed abundances of the 10 most abundant genera recovered by both sequencing methods, with site_type included as a fixed factor and Year–Month (YW) as a random effect [92]. In addition, glmmTMB was applied to evaluate the correlation between amplicon and shotgun sequencing data, using the amplicon abundance of a specific genus as the response variable (Y) and the corresponding shotgun abundance as the predictor (X, fixed factor), while accounting for site_type and Year–Month as random effects. To investigate temporal pattern similarity between amplicon and shotgun sequencing data, dynamic time warping (DTW) distances were calculated using the dtw package [99] after normalization by genus and site type.

Heatmaps were generated using the pheatmap package (v1.0.13) [100] to visualize the relative abundance patterns of fecal-associated taxa known to carry AMR carriers and AMR gene presence. The data were row-scaled (z-score normalized), so that for each taxon (row), the mean relative abundance across samples was centered at zero and values were scaled by the standard deviation [101]. Hierarchical clustering was performed using Euclidean distance and complete linkage for both rows and columns [100]. To evaluate differences in AMR gene presence across site types, a negative binomial generalized linear model was fitted using the *glm.nb* function in the MASS package [102].

### 2.7 Availability of bioinformatic scripts

The Bioinformatic pipeline and data analysis scripts are available here: https://github.com/AAFC-Bioinfo-AAC/snra-metagenomic-statistics-amrvf-ordc/tree/main.

## 3 Results

### 3.1 Relative strength of amplicon and shotgun metagenomics sequencing in detecting fecal-associated bacterial communities

A total of 499 freshwater samples were analyzed by 16S rRNA gene amplicon sequencing, yielding over 21 million high-quality reads (152,076 amplicon sequence variants, ASVs). A subset of 270 (54.1%) was also subjected to shotgun metagenomic sequencing (average 16.6 million reads per sample).

**Fig. 2:**
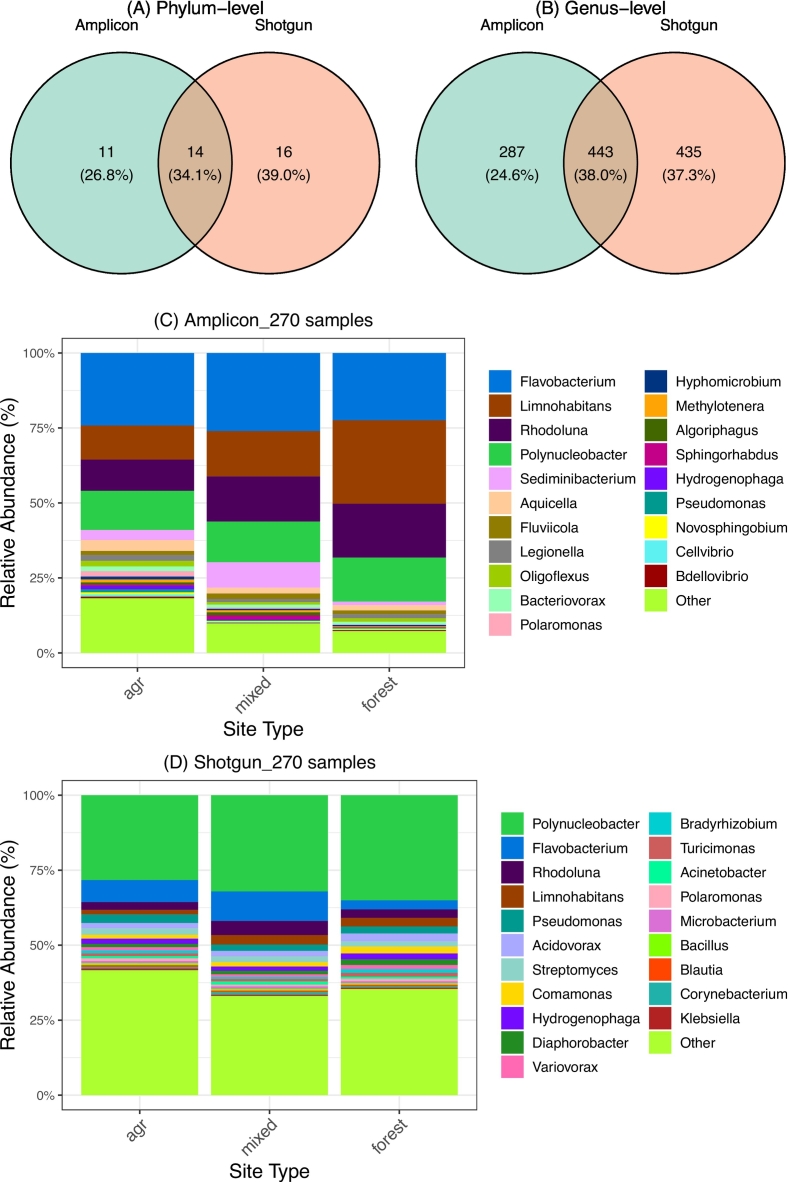
Comparison of fecal-associated bacterial communities identified by amplicon and shotgun metagenomic sequencing. (A-B) Venn diagrams showing overlap of taxa detected at the phylum (A) and genus (B) levels. (C-D) Relative abundance of the 20 most abundant genera in the amplicon (C) and shotgun (D) datasets.

To compare recovery of fecal-associated bacterial taxa, we queried a curated database (Section 2.5) against the paired 270 samples. We identified 730 genera (26 phyla) in the amplicon dataset and 878 genera (30 phyla) in the shotgun dataset. Of these, 14 phyla and 443 genera were shared between the two datasets (Fig. 2A, B). Dominant genera, like *Flavobacterium*, *Limnohabitans*, *Polynucleobacter*, and *Rhodoluna* (Fig. 2C, D), were consistent across methods. However, abundance distributions differed significantly: while non-dominant genera accounted for less than 20% of the relative abundance in amplicon profiles, they contributed over 35% in the shotgun dataset (Fig. 2C, D), indicating that shotgun sequencing recovered a larger proportion of the lower-abundance community fraction in this study.

### 3.2 Fecal-associated bacterial community diversity and pathogen detection across land-use types

#### 3.2.1 Ecological patterns of fecal-associated microbiota based on amplicon sequencing (n = 499)

**Fig. 3:**
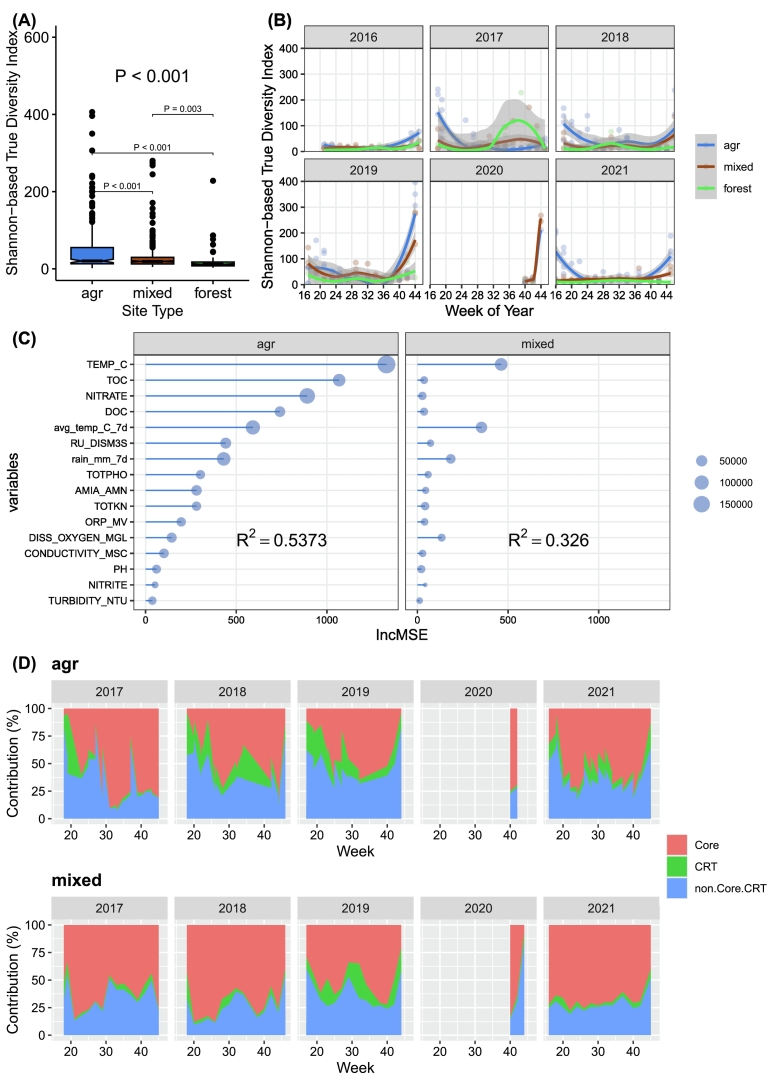
Diversity and dynamics of fecal-associated bacterial communities across land use types. (A) Mean Shannon-Wiener true diversity (SW-TD) index across agricultural drainage ditches, mixed-use, and forested sites. Differences among land-use types were assessed using generalized linear mixed-effects models with Bonferroni-adjusted *P*-values for pairwise contrasts. (B) Seasonal SW-TD dynamics fitted by local polynomial regression (2016–2021). (C) Importance of environmental variables explaining alpha diversity, ranked by random-forest models for agricultural drainage ditches and mixed-use sites. The coefficient of determination (R^2^) for each land-use class is shown. (D) Relative contributions of core and conditionally rare taxa (CRTs) to Bray–Curtis dissimilarity (%) from 2016–2021. Red areas represent core taxa; green areas represent CRTs. Fewer samples were collected in 2020 due to pandemic restrictions. Descriptions of the measured water physicochemical variables are provided in Supplementary Table S1.

Using the 16S rRNA gene amplicon dataset (n = 499), we characterized fecal-associated bacterial communities across different land use classes to help identify potential health risk factors. Across 499 samples, we detected 19,492 ASVs representing 947 fecal-associated genera. Alpha-diversity matrices (Simpson-based true diversity, Shannon-based true diversity, and Chao1 richness) varied significantly with upstream land use (P < 0.05) (Fig. 3A, supplementary Fig. S1). Agricultural drainage ditch sites consistently exhibited the highest diversity, followed by mixed-use and forest sites, with Shannon-based true diversity significantly higher in mixed-use than forested sites (Fig. 3A). Temporal dynamics revealed distinct seasonal peaks in Shannon-based true diversity at the beginning and end of the sampling seasons, particularly in agriculturally dominated ditch sites, except for years with atypical sampling (2016 and 2017) (Fig. 3B). Random Forest (RF) regression identified water temperature as the primary driver of temporal variation in agricultural and mixed-use sites, while total organic carbon (TOC) and nitrate significantly contributed to predictability for agricultural ditches (Supplementary Fig. S2). RF models explained a substantial proportion of diversity variation in agricultural (R^2^ = 0.54) and mixed-use (R^2^ = 0.33) sites but had negligible power at forested sites (Fig. 3C).

We identified 80 core fecal-associated ASVs (70.6% of total abundance) and 1,383 conditionally rare taxa/ASVs (CRTs; 8.6% of abundance), totalling 7.5% of total ASVs across all samples. The remaining (83%) ASVs were predominantly rare (<10 reads). The core microbiota was dominated by *Flavobacterium*, *Limnohabitans_A*, *Polynucleobacter*, and *Rhodoluna*, with *Flavobacterium* including many known zoonotic pathogens. CRT was dominated by *Flavobacterium*, *Legionella*, *Haematospirillum*, *Mycobacterium*, and *Acinetobacter*, all are pathogen-associated genera (See Section 2.5), highlighting the potential public health relevance of this transient but dynamic fraction (supplementary Fig. S3).

Beta diversity analysis indicated that agricultural ditches had significantly lower core but higher CRT contributions to Bray–Curtis dissimilarity compared to mixed-use sites (P < 0.001) (Fig. 3D). Environmental drivers of these fractions differed across different land use classes. RF models explained more variance in agricultural systems (core R^2^ = 0.58; CRT R^2^ = 0.37) than mixed-use sites (core R^2^ = 0.23; CRT R^2^ = 0.08), with dissolved oxygen and TOC emerging as primary predictors (Supplementary Fig. S4). Notably, CRT contributions to fecal-community dynamics in agricultural drainage ditches increased from 6.1% in 2017, 25.9% in 2018, to 20.8% in 2019 (P < 0.05) (Fig. 3D), a trend absent at mixed-use sites. This increase coincided with ditch management (vegetation brushing and dredging); managed sites (SN_20, SN_21) exhibited reduced core contribution (–49.0%) and elevated CRT contribution (+27.9%) in 2019 compared to unmanaged ditches (SN_18, SN_19) (Supplementary Fig. S5). Such observations suggest, either directly or indirectly, physical disturbance and vegetation loss intensified bacterial community turnover in the managed drainage systems.

These pronounced shifts in diversity and CRT contributions within agricultural ditches establish an ecological baseline for evaluating the comparative resolution of shotgun metagenomics in Section 3.2.2.

#### 3.2.2 Cross-validation of fecal-associated microbiota using amplicon and shotgun metagenomics (n = 270)

Using a subset of 270 samples with paired amplicon and shotgun metagenomics datasets, we evaluated cross-method consistency, temporal concordance, and the relative sensitivity for low-abundance or taxonomically complex taxa.

Comparison of the 270 paired samples revealed that while 138 pathogen-associated genera were shared (accounting for >88% of pathogen abundance in both datasets) (supplementary Fig. S6), detection profiles differed for specific taxa. Shotgun metagenomics identified 230 pathogen-associated genera (29.8% of fecal-associated taxa) compared to 157 in the amplicon dataset (37.1%). Among the 10 most abundant genera that were detected by both methods, shotgun sequencing consistently recovered all targets (100% frequency), whereas amplicon detection frequencies varied markedly (e.g., *Corynebacterium* was detected in only 4.4% of samples) (Supplementary Fig. S7).

Both methods detected 18 pathogen-associated bacterial genera listed in the latest CDC zoonotic disease database and 2024 WHO bacterial priority list (Table 1). However, six high-priority genera (*Salmonella, Shigella, Capnocytophaga, Haemophilus, Chlamydia,* and *Streptobacillus*), collectively representing 0.39% of the fecal-associated taxa, were detected only by shotgun sequencing (Table 1). Similarly, *Listeria* presence was corroborated by shotgun sequencing (100% samples) and qPCR (86.9% samples, unpublished data) but was undetected in the amplicon dataset (Supplementary Fig. S8), likely due to primer mismatch (supplementary Table S2).

**Table 1:** Genera containing CDC- and WHO-listed pathogens detected by amplicon and shotgun metagenomic sequencing across different land-use types.

Genera	amplicon						shotgun					
	Relative abundance (%)			Detection frequency			Relative abundance (%)			Detection frequency		
	agr	mixed	forest	agr	mixed	forest	agr	mixed	forest	agr	mixed	forest
*Pseudomonas*	0.993	0.282	0.190	0.602	0.418	0.432	2.810	2.100	2.300	1	1	1
*Rickettsia*	0.246	0.056	0.171	0.585	0.236	0.595	0.079	0.048	0.064	1	1	1
*Acinetobacter*	0.203	0.461	0.015	0.333	0.345	0.216	0.762	1.100	0.719	1	1	1
*Mycobacterium*	0.148	0.166	0.177	0.520	0.573	0.378	0.319	0.353	0.278	1	1	1
*Coxiella*	0.086	0.028	0.014	0.390	0.155	0.270	0.060	0.032	0.057	1	1	1
*Aeromonas*	0.056	0.015	0.016	0.358	0.227	0.270	0.274	0.174	0.196	1	1	1
*Leptospira*	0.023	0.092	0.024	0.236	0.382	0.162	0.082	0.054	0.069	1	1	1
*Escherichia*	0.012	0.001	0.006	0.065	0.018	0.081	0.281	0.246	0.218	1	1	1
*Erysipelothrix*	0.008	0.008	0.000	0.073	0.055	0.000	0.035	0.027	0.023	1	1	1
*Staphylococcus*	0.005	0.000	0.008	0.024	0.009	0.054	0.489	0.330	0.326	1	1	1
*Enterococcus*	0.002	0.001	0.000	0.016	0.018	0.000	0.169	0.136	0.121	1	1	1
*Brucella*	0.001	0.000	0.000	0.016	0.000	0.000	0.073	0.073	0.074	1	1	1
*Neisseria*	0.001	0.000	0.000	0.016	0.000	0.000	0.191	0.162	0.228	1	1	1
*Streptococcus*	0.001	0.001	0.000	0.024	0.027	0.000	0.313	0.238	0.245	1	1	1
*Campylobacter*	0.0002	0.000	0.000	0.016	0.000	0.000	0.254	0.173	0.197	1	1	1
*Borrelia*	0.000	0.000	0.0002	0.008	0.000	0.027	0.037	0.023	0.025	1	1	1
*Bartonella*	0.000	0.001	0.000	0.0000	0.0091	0.0000	0.110	0.102	0.101	1	1	1
*Yersinia*	0.000	0.0001	0.000	0.0000	0.0091	0.0000	0.097	0.080	0.094	1	1	1
*Salmonella*	0.000	0.000	0.000	0.0000	0.0000	0.0000	0.207	0.193	0.126	1	1	1
*Capnocytophaga*	0.000	0.000	0.000	0.0000	0.0000	0.0000	0.081	0.055	0.074	1	1	1
*Haemophilus*	0.000	0.000	0.000	0.0000	0.0000	0.0000	0.076	0.072	0.061	1	1	1
*Chlamydia*	0.000	0.000	0.000	0.0000	0.0000	0.0000	0.065	0.060	0.033	1	1	1
*Shigella*	0.000	0.000	0.000	0.0000	0.0000	0.0000	0.017	0.016	0.032	0.984	0.973	0.946
*Streptobacillus*	0.000	0.000	0.000	0.0000	0.0000	0.0000	0.005	0.003	0.004	1	1	1

Relative abundance (%) represents the proportion of each genus within the total fecal-associated bacterial community. Detection frequency indicates the proportion of samples in which each genus was detected. agr, agricultural drainage ditch sites; mixed, mixed-use sites; forest, forested reference site.

**Fig. 4:**
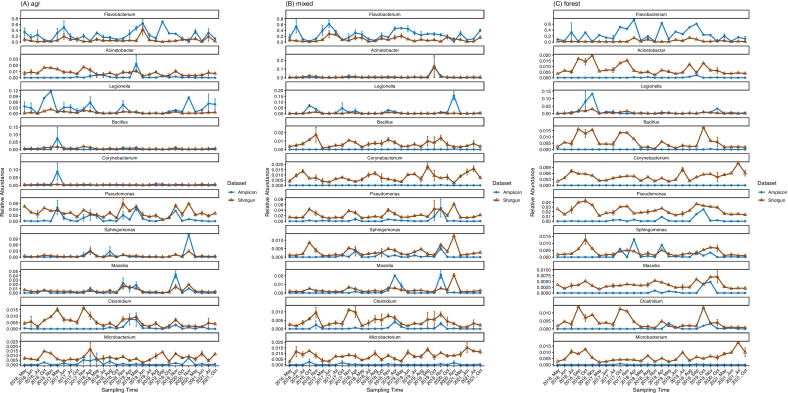
Temporal variation of ten shared genera among the twenty most abundant taxa identified in both sequencing approaches. Mean relative abundances are shown over time for (A) agricultural drainage ditches, (B) mixed-use sites, and (C) forested sites. Error bars represent standard errors.

Ecological resolution also varied between methods. Amplicon profiles showed significant differentiation among different land use classes, with *Clostridium, Massilia, Pseudomonas, Rickettsia, and Sphingomonas* significantly enriched in agricultural ditches (*P* < 0.001) (Fig. 4). Furthermore, *Brucella, Campylobacter, and Neisseria* detection were restricted to agricultural ditches in the amplicon dataset, while *Bartonella* and *Yersinia* were specific to mixed-use sites. *Erysipelothrix* and *Enterococcus* were detected in both agricultural and mixed-use sites but not in the forested reference site (Table 1). In contrast, shotgun profiling detected these genera across most samples (>90% frequency) with no significant abundance differences between site types (Table 1).

Despite these spatial discrepancies, temporal dynamics showed broad concordance. GLMMs revealed strong positive correlations (slope = 0.9-1.2; P < 0.001) for *Flavobacterium* and *Legionella*, as well as moderate correlations (slope < 0.5) for *Sphingomonas* (P < 0.001), *Pseudomonas* (P = 0.001), and *Massilia* (P = 0.004). Dynamic time warping (DTW) confirmed this synchrony, particularly for *Acinetobacter*, which peaked synchronously in October 2019 at mixed-use sites in both datasets (Fig. 4; Supplementary Fig. S9 and S10).

Overall, cross-validation confirmed high concordance for dominant taxa while highlighting a critical trade-off: amplicon profiling resolved stronger ecological differentiation along land-use gradients, whereas shotgun metagenomics provided superior coverage of rare, clinically relevant genera more relevant for functional One Health surveillance.

### 3.3 Prevalence and distribution of resistome and virulome (n = 270)

To characterize reservoirs of antimicrobial resistance and virulence, we analyzed 270 water samples using both short-read metagenomics and metagenome-assembled genomes (MAGs).

**Fig. 5:**
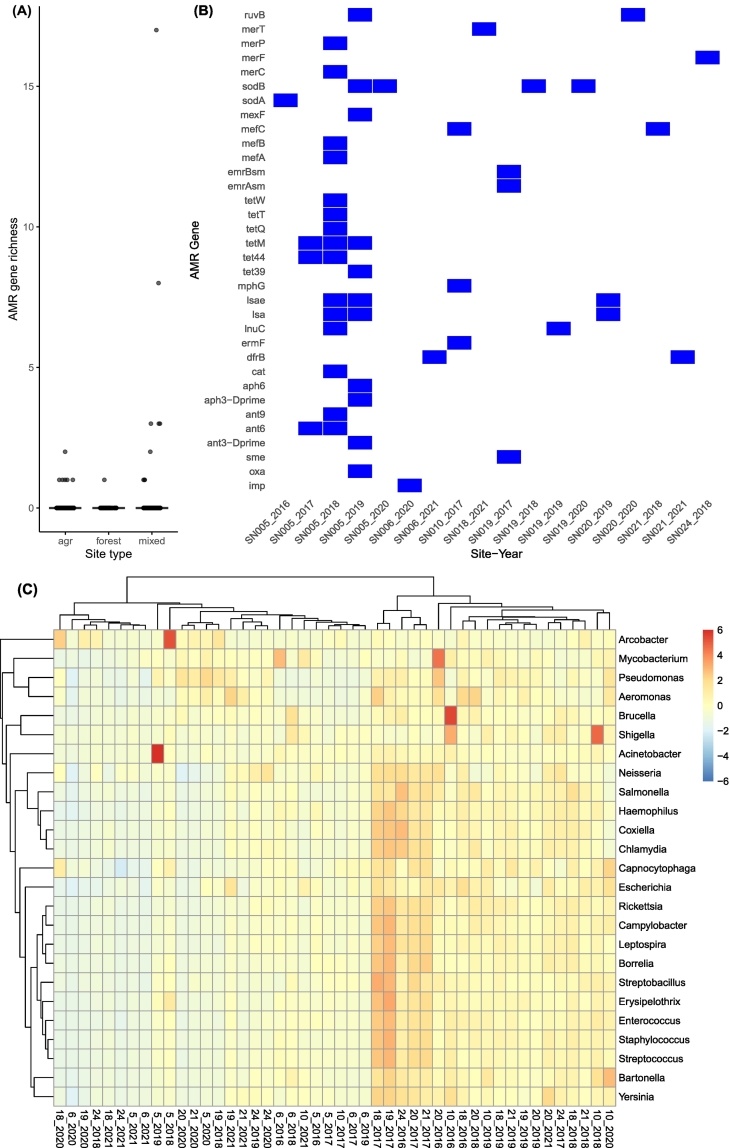
Detection of antimicrobial resistance genes (ARGs) and associated taxa based on short-read shotgun data. (A) AMR gene detection counts across site types. (B) Heatmap showing presence of AMR genes by site and year. (C) Heatmap of genera known to harbor AMR genes across sites and years; colors indicate row-scaled (z-score) relative abundance (red = higher, blue = lower relative to the genus mean).

Short-read metagenomic profiling detected 27 distinct ARGs in 12 samples and 14 VFs in 15 samples (Table 2). Most genes were rare, of which, eight ARGs and one VF (*csrA*) occurred in multiple samples, while the remainder appeared only once (Table 2; Fig. 5). Spatial distributions of these genes were markedly distinct: mixed-use sites were hotspots for resistance, harboring 24 of the 27 detected ARGs (19 specifically at site SN_5 during 2018-2019), whereas agricultural ditches were reservoirs for virulence, containing 13 of the 14 identified VFs (Fig. 5). Consequently, ARG richness was significantly higher at mixed-use sites compared to agricultural ditches (negative binomial model, *P* < 0.05).

**Table 2:** Antimicrobial resistance genes (ARG) and virulence factors (VFs) detected by short-read and metagenome-assembled genome (MAG) analyses from shotgun metagenomic data.

Category	Gene name	Associated drug class/function	AMR relevance/VFs	MAGs #	Taxonomy
I	*imp*	beta-lactamase and carbapenem resistance genes	AMR	-	-
I	*oxa*	beta-lactamase and carbapenem resistance genes	AMR	-	-
I	*sme*	beta-lactamase and carbapenem resistance genes	AMR	-	-
II	*ant3-Dprime*	aminoglycoside resistance genes	AMR	-	-
II	*ant6*	aminoglycoside nucleotidytransferase gene	AMR	-	-
II	*ant9*	aminoglycoside resistance genes	AMR	-	-
II	*aph3-Dprime*	aminoglycoside resistance genes	AMR	-	-
II	*aph6*	aminoglycoside resistance genes	AMR	-	-
II	*dfrB*	plasmid-borne trimethoprim-resistant dihydrofolate reductase	AMR	-	-
II	*ermF*	erythromycin resistance genes	AMR	-	-
II	*lnuC*	transposon-mediated lincosamide nucleotidyltransferase	AMR	-	-
II	*lsa*	resistance to numerous antibiotics, including lincosamides, pleuromutilin, and streptogramins	AMR	-	-
II	*lsae*	resistance to numerous antibiotics, including lincosamides, pleuromutilin, and streptogramins	AMR	-	-
II	*mphG*	macrolide resistance gene	AMR	-	-
II	*mefA*	macrolide efflux pump and transporter genes	AMR	-	-
II	*mefB*	macrolide efflux pump and transporter genes	AMR	-	-
II	*mefC*	macrolide efflux pump and transporter genes	AMR	-	-
III	*cat*	chloramphenicol acetyltransferase gene	AMR	-	-
III	*tet39*	tetracycline resistance genes	AMR	-	-
III	*tet44*	tetracycline resistance gene	AMR	-	-
III	*tetM*	tetracycline resistance gene	AMR	-	-
III	*tetQ*	tetracycline resistance genes	AMR	-	-
III	*tetT*	tetracycline resistance genes	AMR	-	-
III	*tetW*	tetracycline resistance genes	AMR	-	-
not class-specific	*emrAsm*	efflux pump and transporter genes	AMR	-	-
not class-specific	*emrBsm*	efflux pump and transporter genes	AMR	-	-
not class-specific	*mexF*	Part of RND efflux system in *Pseudomonas*	AMR	-	-
not class-specific	*sodA*	multidrug resistance gene	Indirect	-	-
not class-specific	*sodB*	resistance to heavy metals and antimicrobial resistance	Indirect	-	-
not class-specific	*merC*	mercury resistance genes	co-selection link	-	-
not class-specific	*merF*	mercury resistance genes	co-selection link	-	-
not class-specific	*merP*	mercury resistance genes	co-selection link	-	-
not class-specific	*merT*	mercury resistance gene	co-selection link	-	-
not class-specific	*ruvB*	tellurium resistance	co-selection link	-	-
	VFG000863	heat-stable enterotoxin 1	VF		
	VFG001225	twitching motility protein PilG	VF		
	VFG001256	flagellar motor switch protein FliM	VF		
	VFG001855	Hsp60, 60K heat shock protein HtpB	VF		
	VFG002070	type VI secretion system tubule-forming protein VipB	VF		
	VFG010906	carbon storage regulator CsrA	VF		
	VFG013948	type 4 fimbrial biogenesis protein PilZ	VF		
	VFG038176	outer membrane protein OmpA	VF		
	VFG038340	Type III secretion system protein, LcrG-like	VF		
	VFG038346	Type III secretion system gatekeeper subunit	VF		
	VFG038659	flagellar motor switch protein FliN	VF		
	VFG038663	flagellar biosynthesis protein, FliO	VF		
	VFG038876	lateral flagellar basal-body rod protein LfgG	VF		
	VFG043016	purine-binding chemotaxis protein	VF		

MAG-detection					
II	*srmB*	Multi-drug, macrolide, spiramycin resistance gene	AMR	775	CAJAUT01 (Patescibacteria)
II	oqxB	Drug_and_biocide_resistance	AMR	616	Zwartia (Pseudomonadota)
not class-specific	*vmeF*	Multi-biocide RND efflux pump	AMR	17	Algoriphagus (Bacteroidota)
not class-specific	*ileS*	Mupirocin	AMR	530	JALRLI01 (Actinomycetota)
not class-specific	*hpcopA*	copper resistance gene	co-selection link	166	UBA955 (Bacteroidota)
	VFG045688	isoprenyl transferase	VF	209	UBA970 (Bacillota)
	VFG015782	response regulator	VF	369	DMER64 (Bacteroidota)
	VFG047044	glucose-1-phosphate thymidylyltransferase	VF	648	Lacibacter (Bacteroidota)
	VFG010916/VFG010917	two-component system sensor histidine kinase LetS	VF	665	UBA10906 (Pseudomonadota)
	VFG009124	3-oxoacyl-ACP synthase KasB	VF	530	JALRLI01 (Actinomycetota)
	VFG009242	3-oxoacyl-ACP synthase KasB	VF	530	JALRLI01 (Actinomycetota)
	VFG022567	3-oxoacyl-ACP synthase KasB	VF	530	JALRLI01 (Actinomycetota)
	VFG009437	metal-dependent transcriptional regulator	VF	530	JALRLI01 (Actinomycetota)
	VFG009805	response regulator transcription factor	VF	530	JALRLI01 (Actinomycetota)
	VFG009807	response regulator transcription factor	VF	530	JALRLI01 (Actinomycetota)
	VFG009808	response regulator transcription factor	VF	530	JALRLI01 (Actinomycetota)
	VFG024163	response regulator transcription factor	VF	530	JALRLI01 (Actinomycetota)

Antimicrobial resistance genes (ARGs) were categorized according to the importance of their associated antimicrobial drug classes in human medicine in Canada, following the Canadian Integrated Program for Antimicrobial Resistance Surveillance (CIPARS) classification. Category I includes drugs of very high importance, Category II includes drugs of high importance, and Category III includes drugs of moderate importance. “MAG #” indicates the number of metagenome-assembled genomes in which each gene or factor was detected, and “Taxonomy” denotes the corresponding genus-level (phylum) classification when available.

Genome-resolved analysis of 41 medium- to high-quality MAGs provided complementary host-linkage data (Supplementary Table S3). Five bacterial MAGs carried ARGs (Table 2) including *vmeF* (RND efflux, *Algoriphagus*), *srmB* (ABC efflux, CAJAUT01), *oqxB* (multidrug resistance, *Zwartia*), *ileS* (mupirocin resistance, JALRLI01), and *hpcopA* (copper resistance). Notably, these MAG-derived ARGs did not overlap with those found in short-read profiles, highlighting the independent value of each assembly method. Additionally, five MAGs encoded VF genes (Table 2), including loci encoding a multispecies isoprenyl transferase (in MAG 209), response regulator (in MAG 369), a metal-dependent transcriptional regulator (*kasB*; MAG 530), a polysaccharide biosynthesis enzyme *rfbA* (MAG 648), and ATP-binding proteins (MAG 665). These genes represent potential virulence determinants involved in stress response, cell-surface modification, and environmental persistence rather than acute pathogenicity [103].

The detected ARGs spanned multiple antimicrobial classes. According to Canadian categorization of antimicrobial importance, three genes, *oxa* (SN_5, 2019), *imp* (SN_6, 2020), and *sme* (SN_19, 2017), include alleles conferring resistance to Category I antimicrobials of very high importance, including beta-lactams and carbapenems (Fig. 5B). Fourteen ARGs confer resistance to Category II antimicrobials of high importance, including aminoglycosides [*ant(3")-I*, *ant(6)*, *ant(9)*, *aph(3')-I*, *aph(6)*), macrolides (*ermF*, *mphG*, *mefA*, *mefB*, *mefC*), trimethoprim (*dfrB*), and lincosamides (*lnuC*, *lsa*, *lsae*). Ten additional ARGs conferred resistance to Category III antimicrobials of moderate importance, primarily tetracyclines (*tet39*, *tet44*, *tetM*, *tetQ*, *tetT*, *tetW*), chloramphenicol (*cat*), and mupirocin (*ileS*) (Table 2). These genes represent diverse resistance mechanisms, like enzymatic drug modification, target protection, and efflux pumps (Table 2), highlighting the functional breadth of AMR in watershed environments.

Consistent with these patterns, known ARG-carrying bacteria such as *Arcobacter* spp. and *Acinetobacter* spp., were abundant at mixed-use SN_5 across multiple years (Fig. 5C). In additional to canonical ARGs, several metal- and stress-response genes were detected, including components of the mercury resistance operon (*merC*, *merF*, *merP*, *merT*), oxidative-stress enzymes (*sodA*, *sodB*), and the DNA-repair helicase *ruvB*.

Collectively, these results reveal spatially distinct reservoirs: mixed-use sites functioned as ARG hotspots, whereas agricultural ditches served as VF reservoirs. Although overall prevalence was low, the functional diversity captured by combining short-read and MAG analyses reflects complex, site-specific selective pressures. These findings underscore the necessity of integrating gene-level and genome-resolved perspectives to accurately and more holistically assess the ecological persistence and dissemination potential of resistance and virulence traits.

## 4 Discussion

This five-year longitudinal study illustrates how integrated metagenomic surveillance can characterize microbial risk factors in agriculturally influenced watersheds. By combining the scalability of 16S rRNA gene amplicon sequencing with the functional resolution of shotgun metagenomics, we established a complementary framework for tracking fecal-associated taxa, ARGs, and VFs. Beyond method performance, we identified ecological sentinels, specifically conditionally rare taxa (CRTs) and pathogen-associated genera, that link land-use pressures to potential health risk-factors. Collectively, these findings underscore the value of tiered monitoring to characterize environmental exposure pathways within a One Health continuum.

### 4.1 Complementarity of amplicon and shotgun metagenomics in one health surveillance

Evaluating sequencing trade-offs is critical for optimizing One Health surveillance frameworks. While 16S amplicon sequencing offers the scalability required for long-term ecological monitoring [104], shotgun metagenomics offers broader taxonomic and functional resolution necessary to track antimicrobial resistance and virulence potential [26], [42], [105].

In this study, both approaches successfully captured dominant fecal-associated genera, revealing strong temporal concordance for key taxa. Across the five-year dataset, each method detected more than 700 fecal-associated genera, with substantial taxonomic overlap (> 50%) within the core community. These results contrast with earlier reports suggesting poor recovery in shallow shotgun datasets [105], [106], demonstrating that optimized wet-lab workflow and bioinformatics pipelines can effectively profile pathogen-associated communities in complex environmental matrices.

However, differences emerged in ecological sensitivity and spatial resolution. Several genera with pathogenic potential, such as *Microbacterium, Bacillus,* and *Corynebacterium*, exhibited strong seasonal and site-specific patterns in amplicon data but appeared more evenly distributed in the shotgun profiles. These discrepancies likely reflect taxonomic assignment strategies along with amplification bias and sequencing depth [32], [33], [107], [108]. Whole-genome shotgun reads derive from random genomic regions with heterogeneous taxonomic resolution. Lowest Common Ancestor (LCA)-based classifiers such as Kraken/Bracken [109] tend to assign conserved fragments to higher taxonomic levels, leading to taxonomic compression when aggregating across reads. In contrast, 16S amplicon sequencing targets a phylogenetically informative locus with consistent discriminatory power across taxa, enhancing sensitivity to community shifts along environmental gradients. Interestingly, a recent study from Lemonnier et al. [108] demonstrated that extracting 16S or 23S rRNA fragments from shotgun datasets recapitulated the ecological patterns observed in amplicon sequencing, suggesting that discrepancies between approaches largely reflect differences in locus targeting and taxonomic resolution rather than inherent platform limitations.

Conversely, shotgun metagenomics showed higher sensitivity for low-abundance and clinically relevant taxa. Six genera containing CDC- and WHO-listed pathogens (e.g., *Salmonella, Listeria*) were detected exclusively by shotgun sequencing. Methodological evaluation revealed three main drivers for these discrepancies. *In silico* evaluation revealed a distinct asymmetry in primer performance against the missing genera (*Salmonella, Listeria, Capnocytophaga, Haemophilus,* and *Streptobacillus*). While the forward primer (515F-Y) aligned to these targets with moderate fidelity (typically 17/19 bp matches), the reverse primer (926R) exhibited severe mismatches, yielding only non-specific 7–8 bp alignments (Supplementary Table S3). This confirms that reverse primer failure prevented amplification, causing the exclusion of these pathogens from the amplicon dataset despite their presence. Quantitative PCR validation further corroborated the shotgun-based detection of *Listeria* and *Acinetobacter baumannii* [25], [36]. Second, template abundance played a role. All genera exclusive to the shotgun dataset were low-abundance taxa. PCR amplification inherently favors abundant templates, potentially masking rare members even in the absence of severe primer mismatches [110]. This aligns with findings by Durazzi et al. [29], who showed that deep shotgun metagenomics often recovers rare taxa missed by amplicon profiling. Finally, database limitations constrained resolution for specific groups. The 16S rRNA gene cannot reliably distinguish closely related genera; notably, even with the updated Greengenes2 database, *Escherichia* cannot be resolved from *Shigella* due to intrinsic sequence similarity [111]. Collectively, these findings indicate that specific primer mismatches and database constraints, rather than biological factors, account for the detection gaps in the amplicon surveillance.

In summary, these findings support a tiered One Health surveillance strategy. Amplicon sequencing serves as the scalable “ecological baseline” for long-term monitoring of community shifts and sentinel taxa (e.g., CRTs) across broad spatiotemporal scales. Shotgun metagenomics provides the complementary “targeted risk-factor assessment” layer, offering the functional precision needed to characterize resistance reservoirs, virulence factors, and emerging pathogens at high-priority sites. Integrating these approaches balances cost-effective routine monitoring with the diagnostic resolution required for proactive risk assessment in complex agricultural watersheds [29], [106].

### 4.2 Land use and fecal-associated bacterial communities

Fecal-associated bacterial communities were strongly structured by upstream land use, stream function, and seasonal conditions. Agricultural drainage ditches consistently supported higher alpha diversity than mixed-use or forested sites, with distinct early- and late-season peaks. These temporal patterns likely reflect nutrient and bacterial pulses mobilized by snowmelt, surface runoff, and tile drainage [22], [112], [113], [114]. The emergence of water temperature and nitrate as key predictors links bacterial diversity shifts to the seasonal co-mobilization of nutrients and fecal pollution characteristic of agricultural landscapes [115], [116], [117], [118]. Previous work identified livestock manure, wildlife inputs, and septic effluent as key fecal sources at the current study sites [46], [113], [114], [119], [120].

Land use also altered the balance between core and conditionally rare taxa (CRTs). Agricultural ditches exhibited reduced core stability and elevated CRT contributions, indicating heightened responsiveness to episodic contaminant transport. This instability aligns with the specific physical attributes of drainage ditches, namely their rapid hydrological response, small volume, shallow and sometimes intermittent nature, and proximity to fertilized fields [40], [48], [98], [121], as well as their critical function as wildlife corridors [46], [122].

In this agroecosystem, CRTs functioned as a latent reservoir for relatively high-risk taxa, encompassing 53 genera with zoonotic or opportunistic pathogenic potential (12.6% of all pathogen-associated taxa). Of these, only one genus was identified within the core microbiome. However, CRT enrichment is context-dependent and correlative: in pristine systems, rare taxa may fluctuate due to natural environmental variability rather than contamination or management-induced alterations [40], [98], [122], [123]. Therefore, CRTs are best interpreted as ecosystem-specific indicators of instability rather than universal early-warning signals of pollution impact and impairment. Although rare under stable conditions, these taxa contributed disproportionately to community turnover during stress periods [98], [124], [125]. Given that low-abundance taxa can still harbor ARGs and VFs [126], their episodic dominance represents an underrecognized pathway for pathogen persistence and transmission in hydrologically dynamic watersheds [123], [127].

Collectively, land use and hydrology drive fecal-associated bacterial dynamics, establishing agricultural ditches as persistent upstream hotspots [45]. Strong correlations between nitrate and diversity implicate nutrient-laden agricultural runoff/drainage in mobilizing these communities. Consequently, CRTs emerge as ecological sentinels under the One Health framework; monitoring their fluctuations provides a sensitive early-warning mechanism for detecting environmentally engendered risk-factors and pathogen emergence in agricultural watersheds.

### 4.3 Ecological and public health implications of fecal-associated pathogens

Fecal-associated pathogens serve as direct links between land use practices and One Health risk-factors [128], [129]. In this study, agricultural drainage ditches emerged as important sentinel environments, functioning as convergence zones for manure, runoff/drainage, and wildlife inputs. We consistently detected elevated levels of opportunistic genera, such as *Sphingomonas* spp., *Massilia*, *Microbacterium and Clostridium* [130], [131], [132], [133], [134], within these nutrient enriched, hydrologically responsive systems [135], [136].

Spatial patterning further revealed distinct contamination pathways [112]. Livestock-associated taxa such as *Campylobacter* and *Brucella*, wastes from cattle and poultry operations [137], [138], [139], were confined to agricultural ditches [140], [141]. By contrast, *Bartonella* and *Yersinia* appeared exclusively at mixed-use sites, reflecting complex contamination pathways like urban runoff, wildlife sources, and septic leachate more typical of peri-urban landscapes [142], [143], [144], [145]. Many enriched genera, like *Campylobacter*, *Brucella*, *Rickettsia*, and *Acinetobacter*, appear on CDC and WHO priority pathogen lists [85], [86], supporting their use as indicators of anthropogenic pollution. Temporal dynamics reinforced this link, with pathogen richness peaking during snowmelt and high-flow events, indicating transport-driven mobilization.

Although drainage ditches are not typically direct exposure points for humans, they act as persistent upstream reservoirs influencing downstream waters used for recreation, irrigation, and drinking water. Consequently, the detection of WHO- and CDC-priority pathogens highlights the necessity of integrated surveillance, coupling taxonomic precision with functional screening, to monitor upstream hazards before they impact downstream receptors.

### 4.4 Distinct resistome and virulome profiles in agricultural and mixed-use watersheds

Antimicrobial resistance genes (ARGs) and Virulence Factors (VFs) represent latent reservoirs of pathogenic potential bridging environmental and health domains [128], [129]. We observed low prevalent but distinct spatial segregation of these determinants: agricultural drainage ditches were reservoirs for VFs, while mixed-use sites were hotspots for ARGs. These spatial contrasts align with global evidence that resistance and virulence determinants respond to local environmental determinants and shifts [44], [140], [146].

The detection of ARGs at mixed-use sites, although at low frequency, is consistent with the possibility of co-selection [147], [148], [149], [150], [151] arising from intersecting urban and agricultural inputs. Although selective agents such as metals or antibiotics were not directly quantified, the co-occurrence of metal-resistance (e.g., *merC, merP*) and oxidative-stress (e.g., *sodA*, *ruvB*) determinants alongside high-priority β-lactamases and carbapenemase genes (*oxa, imp*) [151], [152], [153] may reflect shared ecological stressors rather than confirmed selective mechanisms. While short-read profiling provides limited host resolution, multi-method integration identified *Acinetobacter* spp. and *Arcobacter* spp. as dominant ARG carriers at the mixed-use site SN_5. Specifically, genome-resolved analysis confirmed *A. baumannii* as a reservoir for blaOXA-type β-lactamase [154], a finding corroborated by qPCR detection of *oxa-51–like* gene in this species at multiple sampling sites included in this study (Supplementary Fig. S11, data and method unpublished). Similarly, the detection of resistance genes such as *cat*, *lnuC*, *lsaE*, and *mefA/B* at SN_5 in 2018 aligns with culture-based isolation of 279 multidrug-resistant *Arcobacter butzleri* strains from the same location [155], establishing a robust link between the resistome profile and viable hosts.

The integration of short-read and genome-resolved (MAG) approaches reveals that each captures distinct dimensions of the resistome and VFs. The minimal overlap between datasets highlights the trade-off between the broad sensitivity of short-read profiling and the genomic context provided by MAGs [25], [156]. MAG-associated VFs were predominantly regulatory or metabolic (e.g., response regulators, *rfbA, KasB*) rather than toxin-encoding. This suggests that environmental reservoirs function primarily as incubators for adaptive traits, enhancing bacterial persistence and stress tolerance [157], [158], [159], which may eventually be co-opted by clinical pathogens [160], [161].

Consequently, we interpret the detection of these low-abundance determinants not as evidence of active disease, but as indicators of latent risk-factor pathways. Their epidemiological relevance is defined by their potential mobility and the presence of compatible hosts within nutrient-enriched, managed and/or critically disturbed environments [162]. The observed co-occurrence of ARGs, VFs, and metal-resistance genes further validates the necessity of dual-platform surveillance to characterize these complex exposure risks [149].

### 4.5 Limitations and future directions

This study offers a longitudinal One Health perspective on fecal-associated microbial dynamics, pathogen occurrence, and resistance reservoirs. However, several methodological and contextual limitations warrant consideration.

First, both sequencing approaches carry inherent biases. Primer-dependent amplification can skew community profiles toward dominant taxa, as observed for *Flavobacterium* (24.7% in amplicon data vs. 7.8% in shotgun profiles) [31], while primer mismatches led to the underrepresentation of specific pathogens (e.g., *Listeria*, *Salmonella*). Conversely, shotgun metagenomics was constrained by sequencing depth (avg. 16.6 million reads/sample in this study vs. > 50–100 million reads per sample in deep sequencing) and high background DNA load [32], [33]. Taxonomic assignment represents an additional limitation. While the Kraken2/Bracken framework used here is computationally efficient, alternative marker-gene strategies (e.g., MetaPhlAn/ChocoPhlAn implemented in HUMAnN) can, in principle, infer species-level presence [163]. However, such inference is strictly limited to reference-covered organisms and fails to capture strain-level or plasmid-borne virulence and AMR determinants [164]. Thus, different tools can yield varying levels of taxonomic resolution and may influence between-sample differentiation, particularly in complex environmental matrices. While a full re-analysis using alternative classifiers or rRNA fragment extraction, as recommended by Lemonnier et al. [108], was beyond the scope of this study, it represents a critical direction for future method comparison. Given these bioinformatic constraints combined with shallow sequencing depth, robust species-level resolution was not feasible. Consequently, classification was restricted to the genus level, and we relied on targeted qPCR assays for species-specific confirmation and quantification.

Second, environmental heterogeneity introduces complexity that challenges the resolution of pulse disturbances. While hydrological variability and land use intensity drive microbial mobilization, bi-weekly sampling intrinsically underrepresents transient, high-impact stressors—including extreme rainfall pulses, storm-driven runoff/discharge, pesticide and manure applications, and point-source discharges like lagoon discharge events [39], [165], [166], [167]. This constraint limits the resolution of fine-scale disturbance responses. Although the current design provided robust long-term coverage, future surveillance should prioritize event-based sampling during hydrologically sensitive periods (e.g., snowmelt, stormflow) to capture the rapid mobilization of pathogens, ARGs, and VFs.

To fully operationalize these ecological insights, surveillance must also expand analytically. Integrating multi-omic approaches (e.g., long-read sequencing, metatranscriptomics) would resolve the strain-level pathogenicity and gene expression gaps identified here. Furthermore, coupling these high-resolution molecular data with Microbial Source Tracking (MST) [168], [169] and Quantitative Microbial Risk Assessment (QMRA) is essential to translate gene detection into more actionable risk probabilities. Embedding this event-driven, multi-omic strategy into watershed management will strengthen the predictive capacity of One Health surveillance, directly informing mitigation policies for sustainable agricultural landscapes.

## 5 Conclusion

This five-year longitudinal study establishes a critical baseline for integrating microbial ecology into One Health surveillance. We demonstrated that a tiered monitoring strategy, coupling the ecological scalability of amplicon sequencing with the functional precision of shotgun metagenomics, is essential for resolving the complex interplay between land use determinants and microbial risk-factors. Biologically, agricultural drainage ditches functioned as persistent upstream reservoirs of elevated diversity and pathogen-associated taxa, where conditionally rare taxa (CRTs) served as sensitive sentinels of environmental change. Conversely, mixed-use watersheds emerged as relative hotspots for antimicrobial resistance, with patterns compatible with potential co-selection in transitional landscapes characterized by varied source contributions. It is suggested to prioritize these high-risk interfaces and shift toward more event-based sampling, or systematic-event hybrid, to capture pulse-driven mobilization. Ultimately, integrating these multi-omic, risk-based approaches into policy/public health frameworks will transform passive environmental monitoring into proactive, evidence-based mitigation of zoonotic and resistance threats.

## Crown copyright

©2026 His Majesty the King in Right of Canada, as represented by the Minister of Agriculture and Agri-Food Canada. Submitted for possible open access publication under the terms and conditions of the Creative Commons Attribution (CC BY) license.

## CRediT authorship contribution statement

**Yichao Shi:** Writing – review & editing, Writing – original draft, Visualization, Methodology, Formal analysis, Conceptualization. **Haley Sanderson:** Writing – review & editing, Methodology. **Jiacheng Chuan:** Writing – review & editing, Methodology. **Izhar U.H. Khan:** Writing – review & editing, Data curation. **Mark Sunohara:** Writing – review & editing, Data curation. **Emilia Craiovan:** Writing – review & editing, Data curation. **David R. Lapen:** Writing – review & editing, Data curation, Funding acquisition. **Moussa Diarra:** Writing – review & editing. **Wen Chen**: Writing – review & editing, Supervision, Methodology, Funding acquisition, Conceptualization.

## Funding

The author(s) declare that financial support was received for the research, authorship, and/or publication of this article. The study was financially funded by the Government of Canada’s Environmental Change Onehealth Observatory Project (ECO2, J-002305), the Genomics Research and Development Initiative-Metagenomics Based Ecosystem Biomonitoring (GRDI-EcoBiomics, J-001263). Salary of bioinformatician was supported by the SCAP-ASC-18 Poultry Cluster Activity 5A - Microbiome in Poultry Production Environment as a Function of Climate and Alternative Use of Litter (J-003360).

## Declaration of competing interest

The authors declare that the research was conducted in the absence of any commercial or financial relationships that could be construed as a potential conflict of interest.

## Data Availability

The raw paired-end 16S rRNA gene amplicon sequencing data have been deposited in the NCBI Sequence Read Archive (SRA), under the Bioproject accession PRJNA858176 (https://www.ncbi.nlm.nih.gov/bioproject/PRJNA858176), Biosample accessions SAMN29671977–2598 and SAMN56369704–56369930. The raw shotgun sequencing data in FASTQ format are accessible from the European Nucleotide Archive under the Study accession PRJEB102733, with the samples accessions ERS27328153–ERS27328689.

## Appendix A Supplementary data

Supplementary material Image 1
