# Supplementary material for: Dual-platform metagenomic surveillance distinguishes pathogen and resistome hotspots across agricultural and mixed-use watersheds
Source: One Health. 2026 Mar 8;22:101384. doi: 10.1016/j.onehlt.2026.101384 (PMC13015239; doi:10.1016/j.onehlt.2026.101384)
Supplement: Supplementary file 1 — Supplementary material [file mmc1.docx]

**Dual-Platform Metagenomic Surveillance Distinguishes Pathogen and Resistome Hotspots Across Agricultural and Mixed-Use Watersheds**

Yichao Shi^a^, Haley Sanderson^b^, Jiacheng Chuan^c^, Izhar U.H. Khan^a^, [Mark Sunohara](https://link.springer.com/article/10.1186/s12866-023-02755-7#auth-Mark-Sunohara-Aff1)^a^, [Emilia Craiovan](https://link.springer.com/article/10.1186/s12866-023-02755-7#auth-Emilia-Craiovan-Aff1)^a^,  David R. Lapen^a^, Moussa Diarra^d^, and Wen Chen^a,e^*

^a^ Ottawa Research & Development Centre, Science & Technology Branch, Agriculture & Agri-Food Canada (AAFC), 960 Carling Avenue, Ottawa, ON K1A 0C6, Canada

^b^ Biological Informatics Center of Excellence, Information Systems Branch, AAFC, 107 Science Pl, Saskatoon, SK S7N 5E2, Canada

^c^ The Charlottetown Laboratory, Canadian Food & Inspection Agency (CFIA), 93 Mt Edward Rd, Charlottetown, PEI C1A 5T1, Canada

^d^ Guelph Research and Development Centre, AAFC, 93 Stone Rd W, Guelph, ON N1G 5C9, Canada

^e^ Department of Biology, University of Ottawa, Marie-Curie Private, Ottawa, ON, K1N 9A7, Canada

*Corresponding author at Ottawa Research & Development Centre, Science & Technology Branch, Agriculture & Agri-Food Canada (AAFC), 960 Carling Avenue, Ottawa, ON K1A 0C6, Canada.

E-mail address: [wen.chen@agr.gc.ca](mailto:wen.chen@agr.gc.ca)

**Supplementary documents**

**S.1 Supplemental Tables**

**Supplementary Table S1.** Definitions of land use, water physicochemical, and environmental variables in this study.

| Variable name | Variable description (unit) |
| --- | --- |
| site_type | Sampling site land use class (Agriculture, Mixed, Forest) |
| TEMP_C | Temperature read with the YSI mini sonde in water (°C) |
| PH | pH read with the YSI mini sonde in water |
| CONDUCTIVITY_MSC | Water specific conductivity read with YSI mini sonde in water. (mS cm^-1^) |
| DISS_OXYGEN_MGL | Dissolved oxygen read with YSI mini sonde in water (mg L^-1^) |
| ORP_MV | Electronic measurement of oxidation-reduction potential (mV) |
| TURBIDITY_NTU | Turbidity measurement with YSI mini sonde in Nephelometric Turbidity Units (NTU) |
| AMIA_AMN | NH_3_, NH_4_^+^ concentration (mg L^-1^) |
| NITRITE | Nitrite concentration (mg L^-1^) |
| NITRATE | Nitrate concentration (mg L^-1^) |
| TOTKN | Total Kjeldahl Nitrogen (mg L^-1^) |
| TOTPHO | Total phosphorus concentration (mg L^-1^) |
| DOC | Dissolved organic carbon concentration (mg L^-1^) |
| TOC | Total organic carbon concentration (mg L^-1^) |
| RU_DISM3S | Discharge of Castor River at Russell station (02LB006), daily mean water discharge. (m3 s-1) |
| rain_mm_7d | Total rainfall recorded at WEBs meteorological station over 7 days prior, including sampling date. (mm) |
| avg_temp_C_7d | Average temperature recorded at WEBs meteorological station over 7 days prior, including sampling date. (°C) |

**Supplementary Table S2.** In Silico BLASTn Evaluation of Primer Coverage and Specificity Against Reference 16S rRNA Sequences

| **Genus** | **Taxa (n)** | **Reference sequences (n)** | **Forward primer (19 bp)** | **Reverse primer (18 bp)** |
| --- | --- | --- | --- | --- |
| Capnocytophaga | 11 | 60 | 92% taxa matched; <90% identity; 2 mismatches; E = 0.008 | Only 2 taxa aligned; 7–8 bp match; E = 513 |
| Haemophilus | 14 | 433 | 42% taxa matched; <90% identity; 2 mismatches; E = 0.008 | 77% taxa aligned; mostly 8 bp match; E = 108 |
| Listeria | 16 | 166 | 24% taxa matched; <90% identity; 2 mismatches; E = 0.008 | 7–8 bp match only; E = 513 |
| Salmonella | 8 | 89 | 100% taxa matched; <90% identity; 2 mismatches; E = 0.008 | No significant alignment |
| Streptobacillus | 4 | 8 | 100% taxa matched; <90% identity; 2 mismatches; E = 0.008 | No significant alignment |

**Supplementary Table S3.** Quality and genomic characteristics of metagenome‑assembled genomes (MAGs) recovered from shotgun metagenomic sequencing. Metrics include completeness, contamination, coding density, contig N50, average gene length, estimated genome size, GC content, and total predicted coding sequences.

| NO# | MAGs # | Completeness (%) | Contamination (%) | Completeness_Model_Used | Coding_Density | Contig_N50 | Average_Gene_Length | Genome_Size | GC_Content | Total_Coding_Sequences |
| --- | --- | --- | --- | --- | --- | --- | --- | --- | --- | --- |
| 1 | 14 | 92.51 | 1.3 | Gradient Boost (General Model) | 0.902 | 57737 | 279.41 | 773972 | 0.41 | 834 |
| 2 | 166 | 100 | 2.98 | Gradient Boost (General Model) | 0.945 | 200289 | 372.92 | 2426813 | 0.46 | 2054 |
| 3 | 171 | 71.98 | 6.9 | Gradient Boost (General Model) | 0.903 | 59639 | 213.62 | 1194109 | 0.41 | 1695 |
| 4 | 23 | 56.77 | 3.92 | Gradient Boost (General Model) | 0.898 | 206968 | 211.18 | 873702 | 0.45 | 1247 |
| 5 | 240 | 60.11 | 0.69 | Gradient Boost (General Model) | 0.902 | 5642 | 263.66 | 989926 | 0.59 | 1133 |
| 6 | 283 | 83.7 | 0.23 | Gradient Boost (General Model) | 0.91 | 16793 | 313.56 | 955794 | 0.39 | 926 |
| 7 | 289 | 62.43 | 9.57 | Gradient Boost (General Model) | 0.939 | 24062 | 258.17 | 1740621 | 0.39 | 2119 |
| 8 | 291 | 55.75 | 4.73 | Gradient Boost (General Model) | 0.933 | 52040 | 192.44 | 772117 | 0.46 | 1253 |
| 9 | 355 | 53.56 | 4.17 | Gradient Boost (General Model) | 0.96 | 17431 | 231.79 | 896938 | 0.41 | 1246 |
| 10 | 367 | 54.14 | 9.45 | Gradient Boost (General Model) | 0.947 | 10065 | 186.49 | 1767817 | 0.61 | 3006 |
| 11 | 39 | 53.13 | 1.21 | Gradient Boost (General Model) | 0.935 | 12034 | 198.12 | 608061 | 0.5 | 962 |
| 12 | 400 | 57.74 | 9.66 | Gradient Boost (General Model) | 0.963 | 8502 | 404.84 | 2464247 | 0.39 | 1956 |
| 13 | 42 | 73.83 | 0.53 | Gradient Boost (General Model) | 0.932 | 8118 | 247.79 | 707509 | 0.47 | 891 |
| 14 | 524 | 62.27 | 8.86 | Gradient Boost (General Model) | 0.927 | 49896 | 180.76 | 825419 | 0.49 | 1421 |
| 15 | 616 | 58.76 | 6.44 | Gradient Boost (General Model) | 0.933 | 7459 | 294.27 | 1527294 | 0.54 | 1618 |
| 16 | 731 | 56.32 | 7.77 | Gradient Boost (General Model) | 0.956 | 22426 | 182.86 | 1049496 | 0.36 | 1845 |
| 17 | 755 | 57.44 | 1.69 | Neural Network (Specific Model) | 0.92 | 6224 | 257.76 | 570437 | 0.41 | 681 |
| 18 | 96 | 56.09 | 4.8 | Gradient Boost (General Model) | 0.924 | 347463 | 197.49 | 864974 | 0.42 | 1360 |
| 19 | 128 | 55.99 | 8.41 | Gradient Boost (General Model) | 0.967 | 15939 | 327.29 | 1754147 | 0.45 | 1733 |
| 20 | 173 | 73.34 | 3.82 | Gradient Boost (General Model) | 0.897 | 86896 | 226.44 | 630951 | 0.43 | 838 |
| 21 | 209 | 50.98 | 0.33 | Gradient Boost (General Model) | 0.945 | 7638 | 285.79 | 554845 | 0.39 | 614 |
| 22 | 271 | 55.54 | 8.66 | Gradient Boost (General Model) | 0.958 | 10569 | 471.95 | 2247858 | 0.42 | 1523 |
| 23 | 530 | 74.26 | 0.2 | Neural Network (Specific Model) | 0.941 | 265673 | 326.95 | 1287027 | 0.57 | 1238 |
| 24 | 549 | 51.67 | 2.51 | Gradient Boost (General Model) | 0.903 | 54799 | 354.74 | 1238884 | 0.33 | 1053 |
| 25 | 589 | 66.47 | 9 | Gradient Boost (General Model) | 0.939 | 32508 | 293.39 | 1476163 | 0.37 | 1582 |
| 26 | 647 | 67.93 | 0.83 | Gradient Boost (General Model) | 0.844 | 8327 | 324.55 | 4407903 | 0.36 | 3825 |
| 27 | 648 | 96.95 | 2.39 | Gradient Boost (General Model) | 0.902 | 19857 | 323.56 | 4544177 | 0.41 | 4231 |
| 28 | 715 | 92.87 | 0.96 | Gradient Boost (General Model) | 0.895 | 33606 | 286.64 | 600537 | 0.42 | 626 |
| 29 | 17 | 92.25 | 0.51 | Neural Network (Specific Model) | 0.896 | 19783 | 312.57 | 2674255 | 0.45 | 2559 |
| 30 | 188 | 64.38 | 7.31 | Gradient Boost (General Model) | 0.311 | 5373 | 78.60 | 9499219 | 0.34 | 12688 |
| 31 | 369 | 97.74 | 0.74 | Neural Network (Specific Model) | 0.948 | 111473 | 354.81 | 2368871 | 0.49 | 2118 |
| 32 | 401 | 54.73 | 6.12 | Gradient Boost (General Model) | 0.977 | 7417 | 1236.91 | 3905454 | 0.57 | 1030 |
| 33 | 47 | 73.03 | 2.83 | Neural Network (Specific Model) | 0.872 | 6763 | 253.30 | 565068 | 0.41 | 649 |
| 34 | 471 | 55.12 | 8.63 | Gradient Boost (General Model) | 0.91 | 53348 | 260.23 | 1439596 | 0.47 | 1681 |
| 35 | 604 | 58.69 | 9.05 | Gradient Boost (General Model) | 0.934 | 8179 | 158.98 | 963332 | 0.49 | 1905 |
| 36 | 749 | 67.34 | 8.14 | Gradient Boost (General Model) | 0.955 | 14199 | 206.25 | 1098352 | 0.44 | 1702 |
| 37 | 797 | 89.49 | 1.9 | Gradient Boost (General Model) | 0.805 | 14870 | 299.91 | 1005130 | 0.46 | 902 |
| 38 | 818 | 92.81 | 0.38 | Gradient Boost (General Model) | 0.931 | 51527 | 306.74 | 1602915 | 0.4 | 1625 |
| 39 | 191 | 56.04 | 5.13 | Gradient Boost (General Model) | 0.935 | 23103 | 169.82 | 780134 | 0.46 | 1443 |
| 40 | 665 | 88.18 | 3.34 | Neural Network (Specific Model) | 0.911 | 155602 | 316.68 | 2514842 | 0.41 | 2416 |
| 41 | 775 | 97.35 | 0.97 | Gradient Boost (General Model) | 0.882 | 37514 | 275.28 | 785002 | 0.51 | 840 |

**S.2 Supplementary Figures**

**
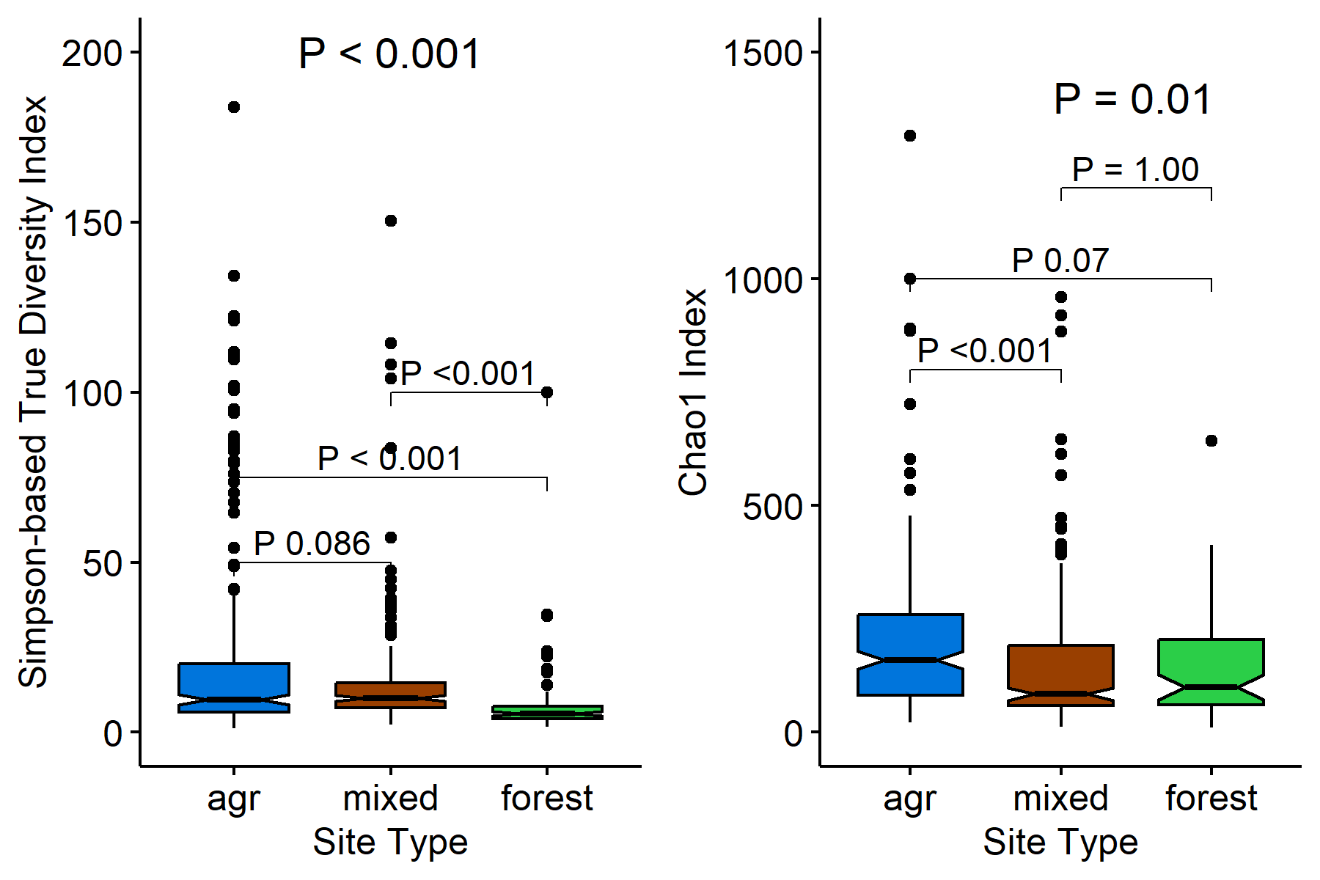
**

**Supplementary Fig. S1.** Mean alpha diversity of fecal-associated bacterial communities measured using the Simpson-based true diversity and Chao1 richness index. Differences among agricultural drainage ditches (agr), mixed-use sites (mixed), and the forested reference site (forest) were evaluated using generalized linear mixed-effects models (GLMMs). Pairwise comparisons were conducted using *emmeans* with Holm-adjusted P-values.

**
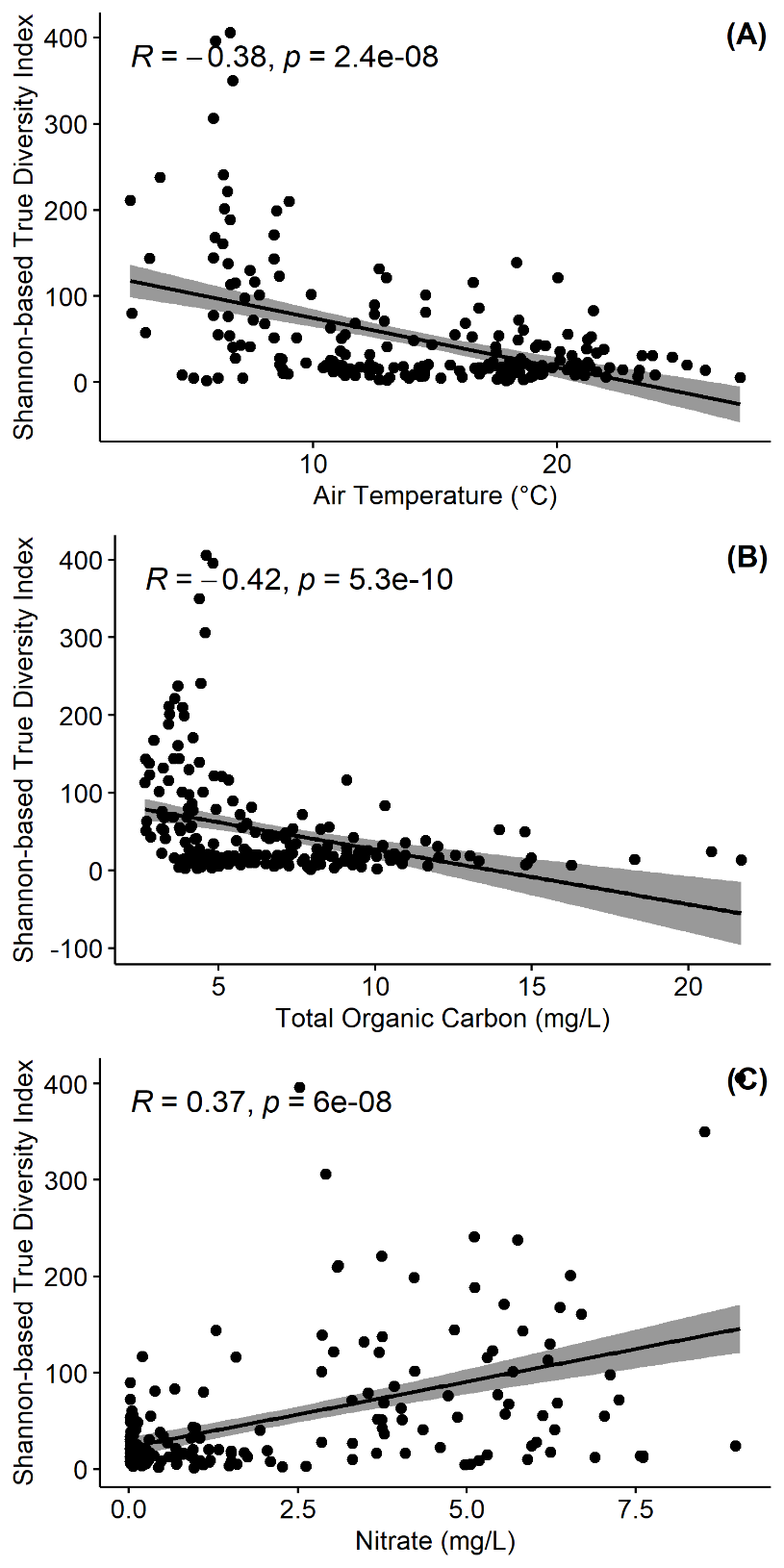
**

**Supplementary Fig. S2.** Spearman’s rank correlations between Shannon-based true diversity and (A) air temperature, (B) total organic carbon, and (C) nitrate concentration. Shaded areas indicate 95% confidence intervals, and significance levels are reported as P values.


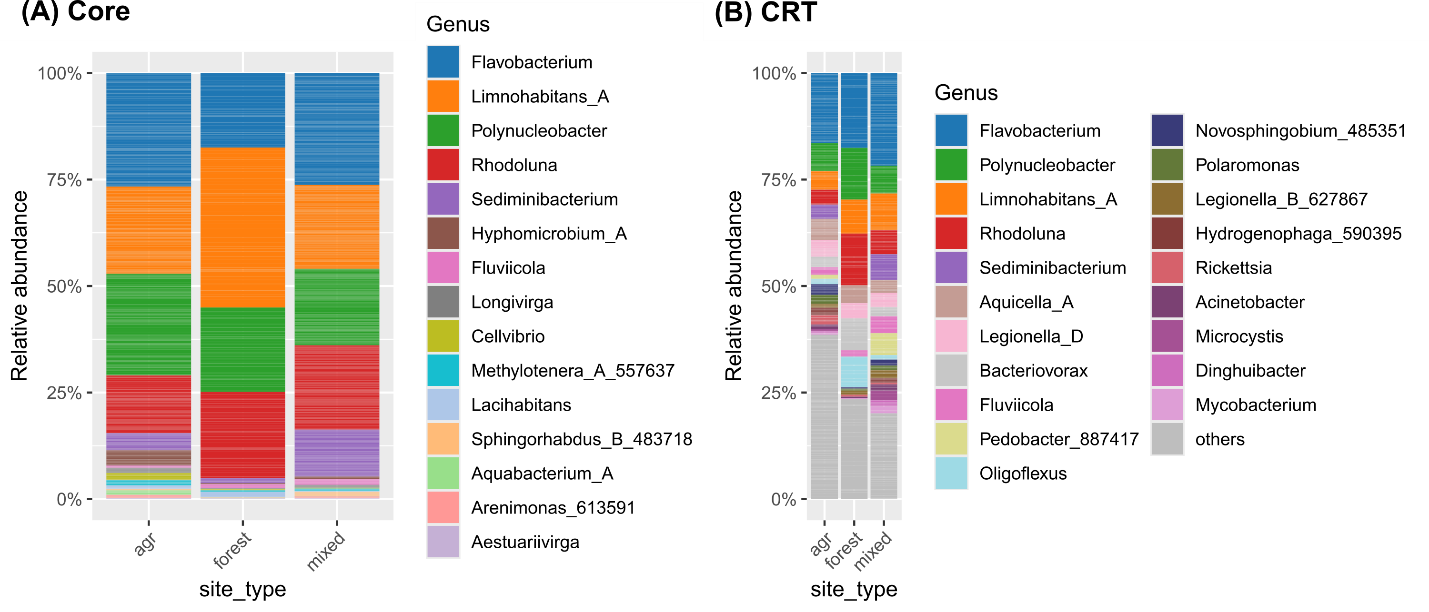


**Supplementary Fig. S3.** Relative abundance of the 20 most abundant genera within (A) the core microbiome and (B) conditionally rare taxa (CRT) across agricultural drainage ditches (agr), mixed-use sites (mixed), and the forested site (forest). Core and CRT members were defined across all sampling sites and years (n = 499).


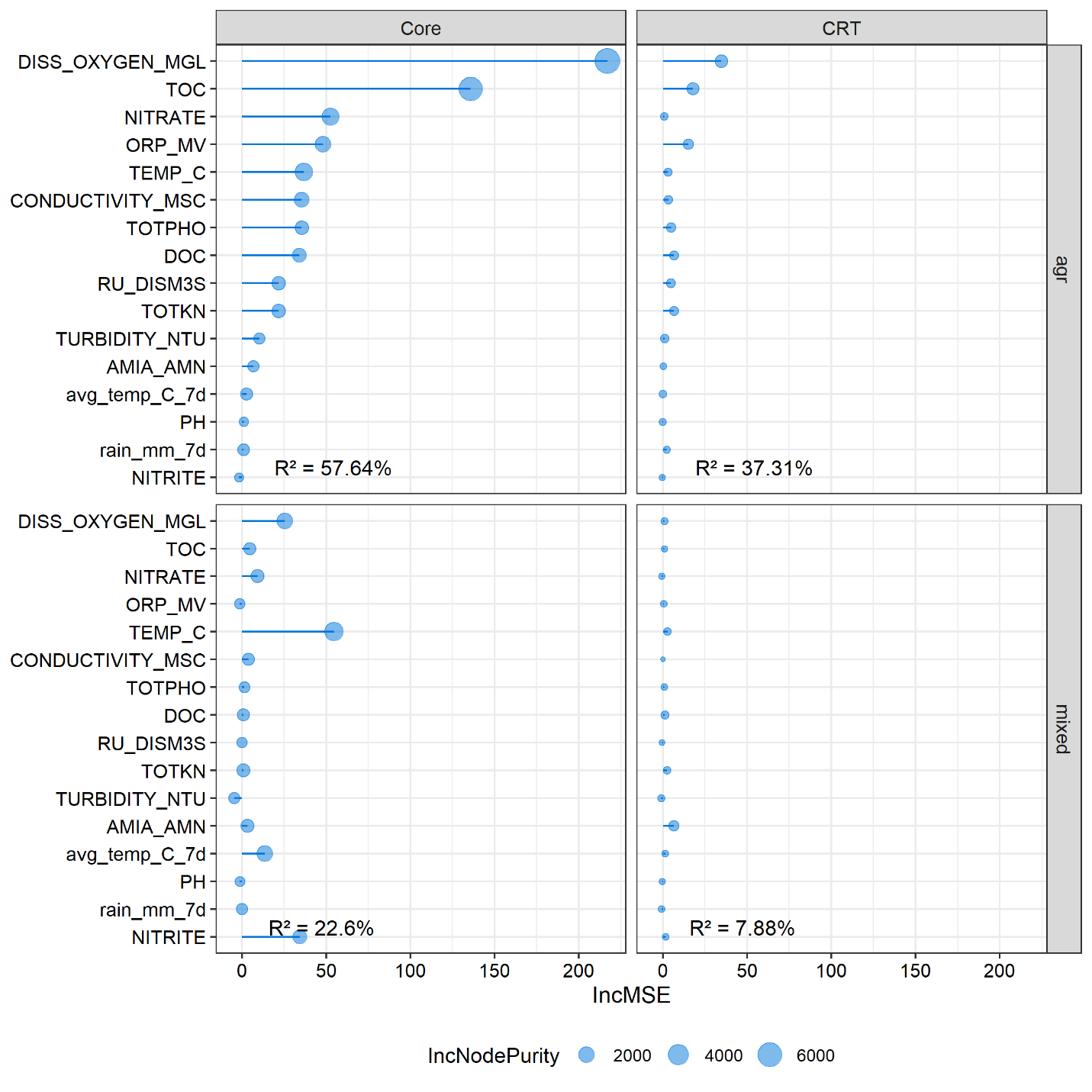


**Supplementary Fig. S4.** Importance of environmental variables influencing the relative contributions of (left) core taxa and (right) conditionally rare taxa (CRT) to the overall Bray–Curtis dissimilarity (%) of fecal-associated bacterial communities. Random forest regression models were constructed for agricultural drainage ditches (top) and mixed-use sites (bottom). Variables are ranked by importance, expressed as the increase in mean squared error (IncMSE), based on the core contribution model for agricultural drainage ditches. The coefficient of determination (R²) is reported for each model.


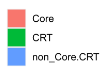

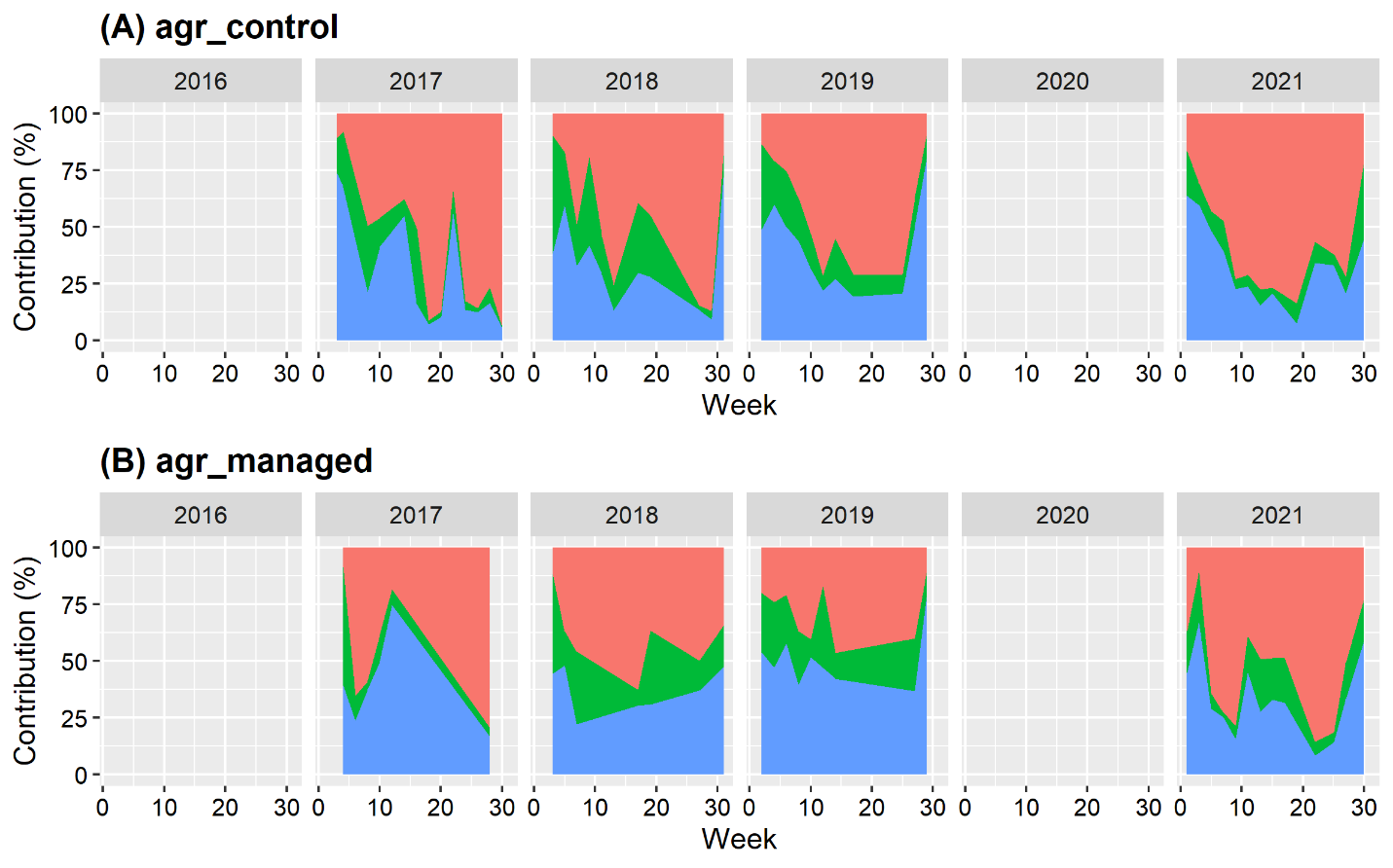


**Supplementary Fig. S5.** Area plots showing the relative contributions of core and conditionally rare taxa (CRT) to the overall Bray–Curtis dissimilarity (%) of fecal-associated bacterial communities from 2016–2021 at agricultural drainage ditch sites. Panels represent (A) unmanaged (control) sites and (B) managed sites. Red areas indicate core taxa dynamics, while green areas indicate CRT dynamics. Data for 2016 and 2020 were unavailable due to insufficient replicates.


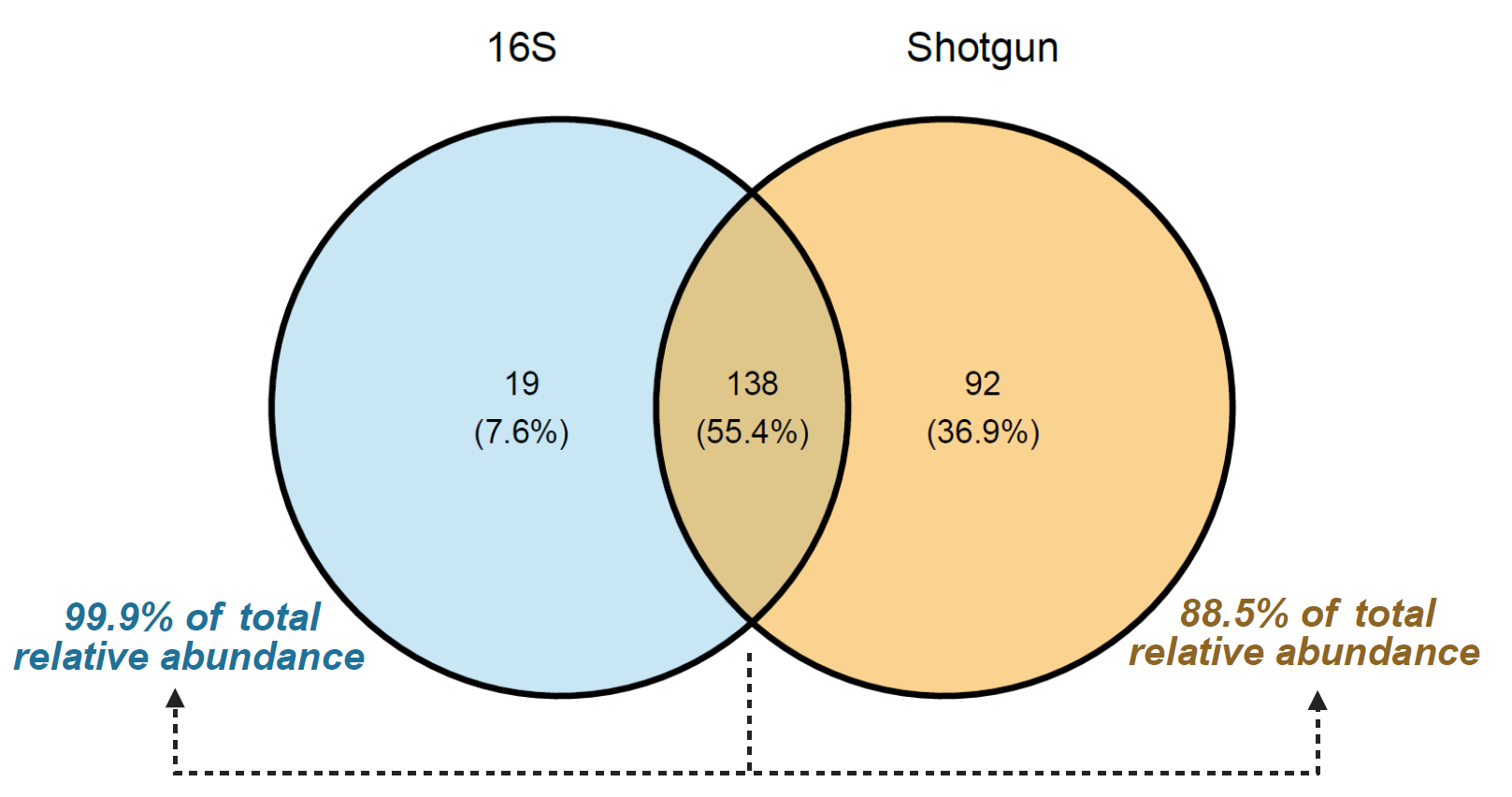


**Supplementary Fig. S6.** Venn diagrams showing the overlap of genera containing potential pathogens detected by amplicon (16S rRNA gene) and shotgun metagenomic sequencing. The proportional contributions of shared genera to the overall pathogen-associated community are shown for each sequencing approach.


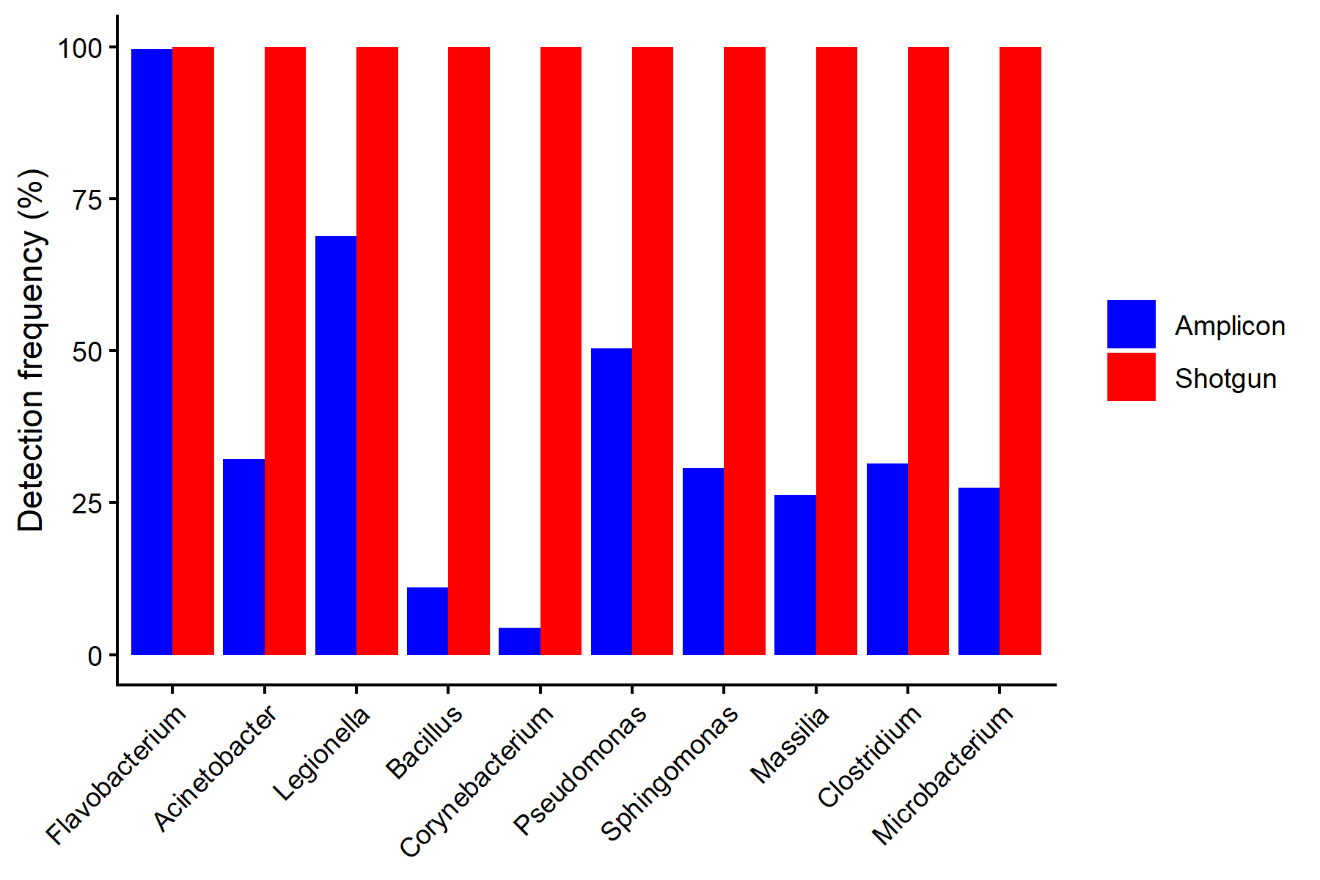


**Supplementary Fig. S7.** Bar plot showing the detection frequency (%) of 10 genera among the 20 most abundant taxa identified in both amplicon and shotgun metagenomic datasets. Detection frequency represents the proportion of samples in which each genus was observed.


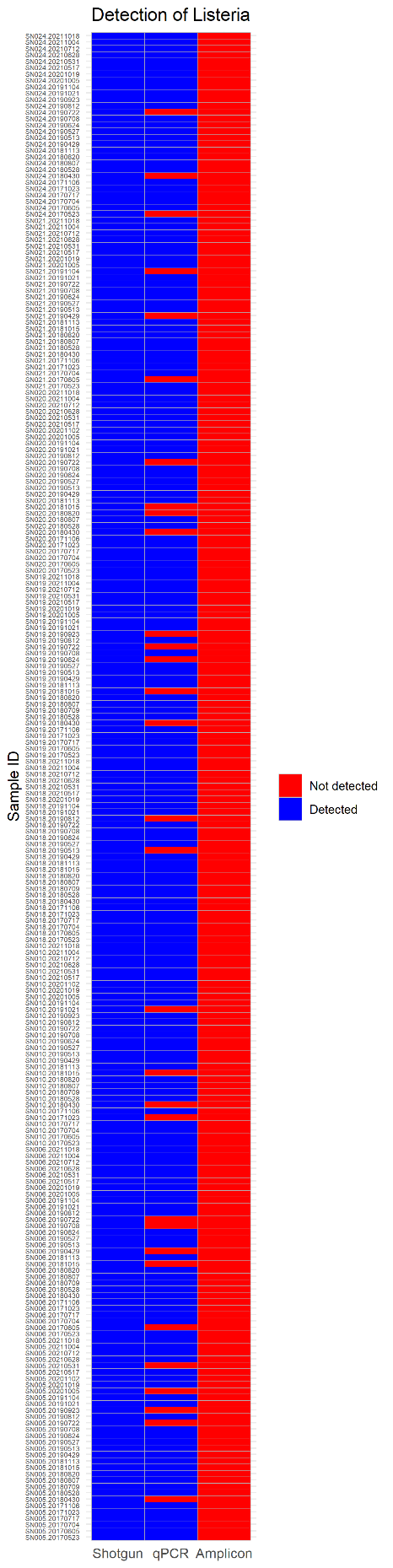


**Supplementary Fig. S8.** Comparison of *Listeria* detection across three methods: amplicon (16S rRNA gene) sequencing, shotgun metagenomics, and quantitative PCR (qPCR). Detection frequency represents the proportion of samples in which Listeria was identified by each method.


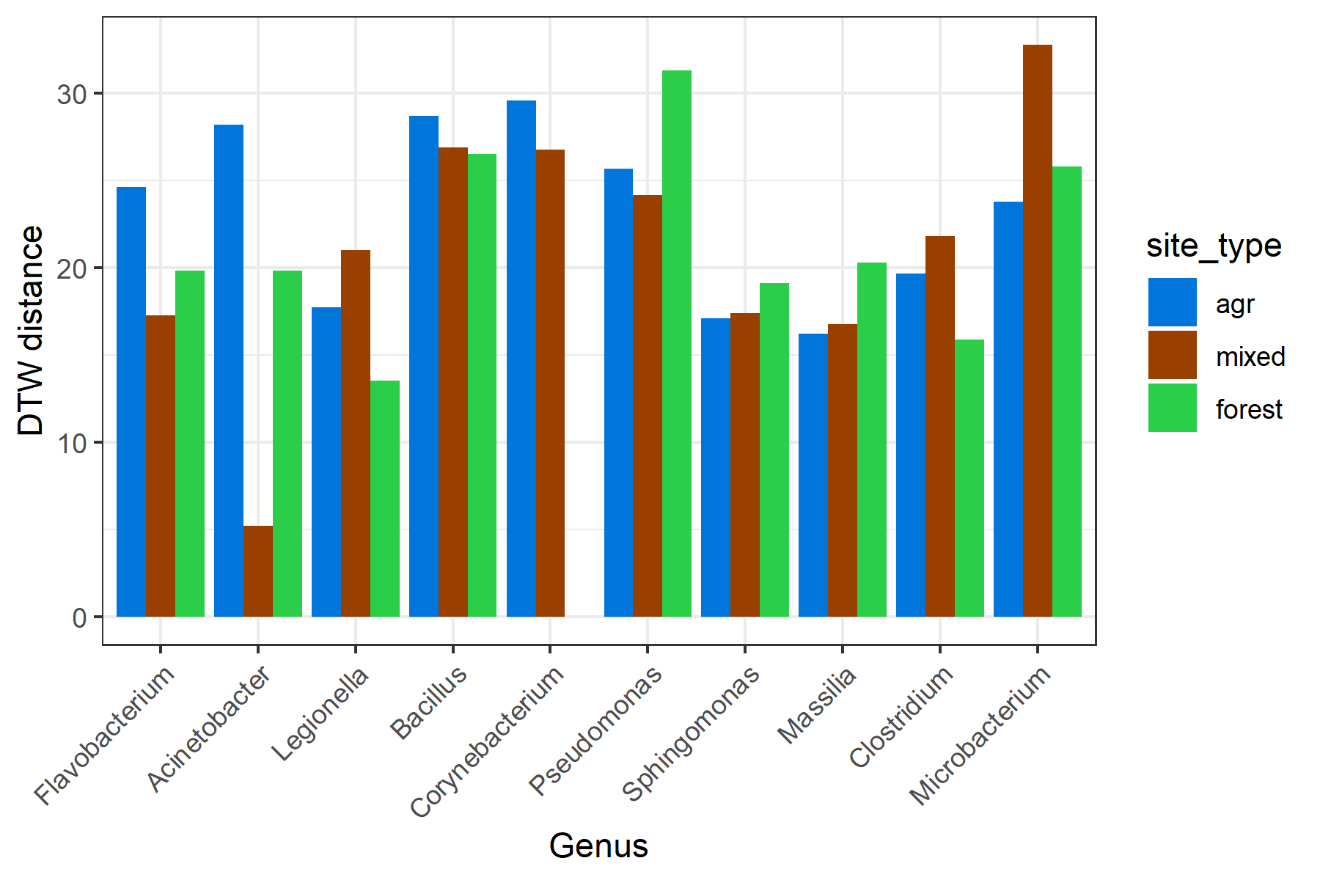


**Supplementary Fig. S9.** Dynamic Time Warping (DTW) distances between amplicon- and shotgun-based relative abundance profiles for 10 genera among the 20 most abundant taxa. DTW distances are shown for agricultural drainage ditch sites (agr), mixed land use sites (mixed), and the forested reference site (forest).


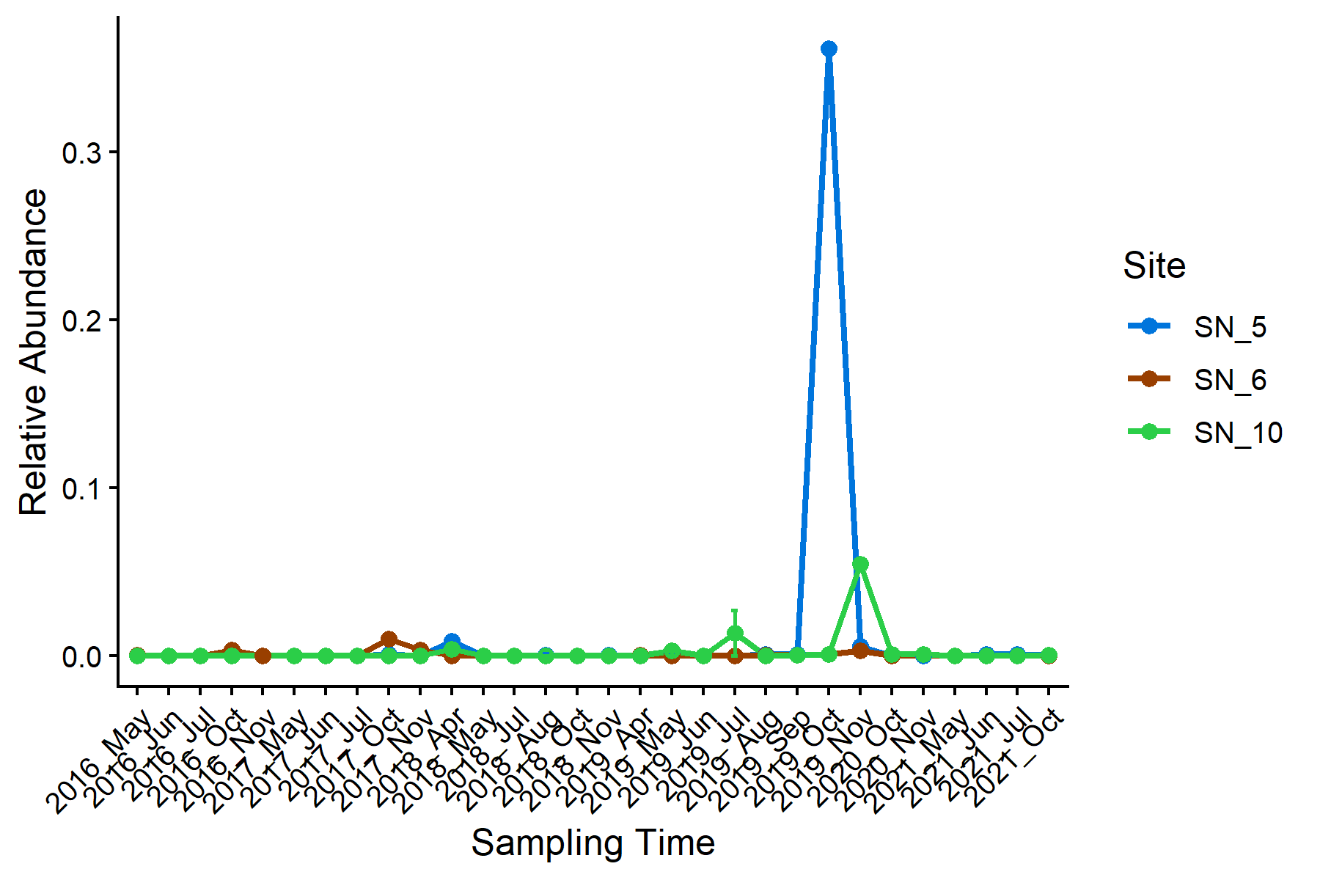


**Supplementary Fig. S10.** Temporal variation in the relative abundance of *Acinetobacter* at individual mixed-use sites. Error bars represent the standard error of the mean for each monthly sampling period.


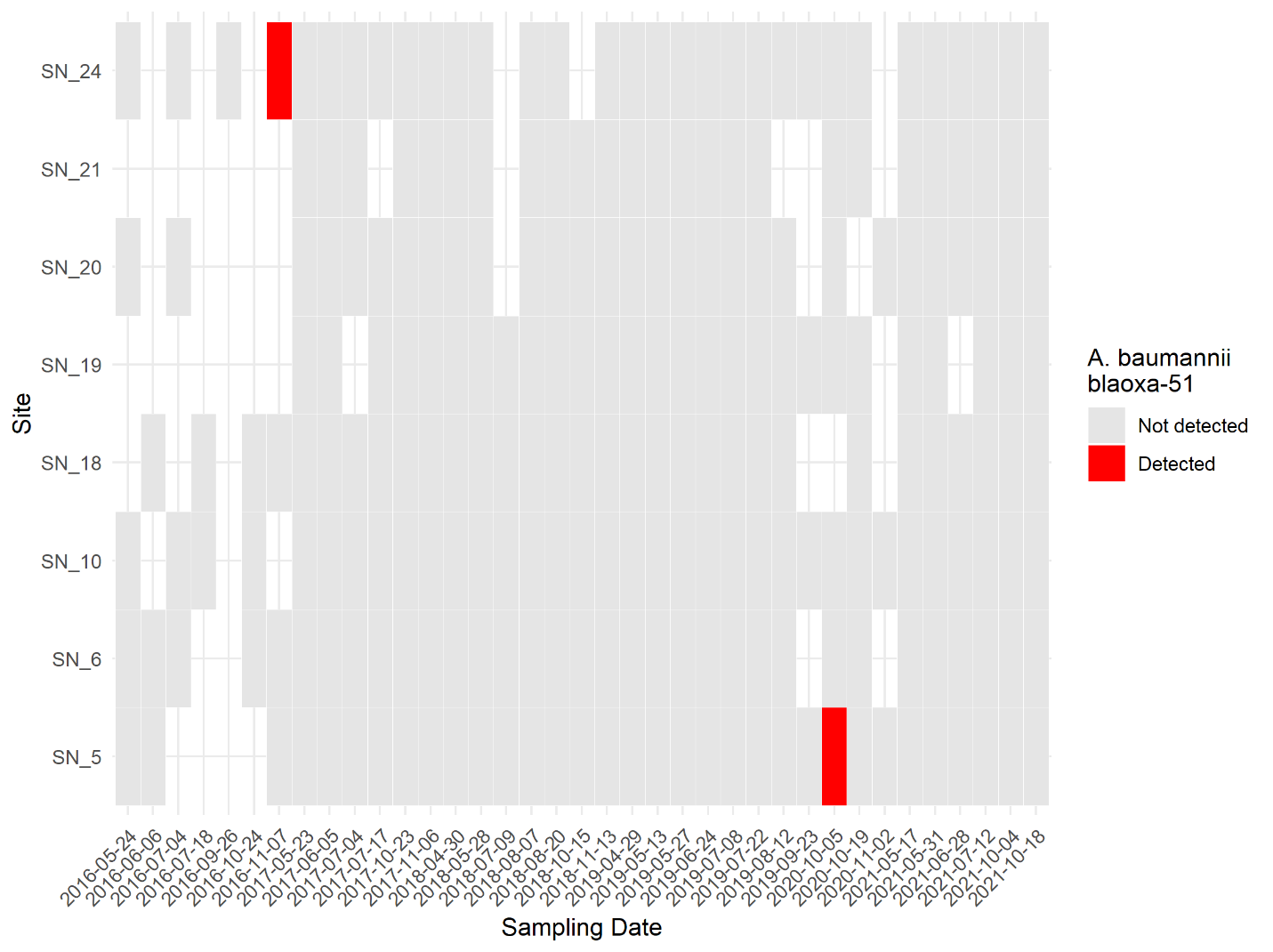


**Supplementary Fig. S11.** Heatmap showing *Acinetobacter baumannii* carrying the *blaOXA-51* gene across sites and sampling dates, based on qPCR detection. Red color indicates the gene was detected in *A. baumannii* in the corresponding sample.
